# A preliminary examination of bacterial, archaeal, and fungal communities inhabiting different rhizocompartments of tomato plants under real-world environments

**DOI:** 10.1038/s41598-019-45660-8

**Published:** 2019-06-26

**Authors:** Shin Ae Lee, Yiseul Kim, Jeong Myeong Kim, Bora Chu, Jae-Ho Joa, Mee Kyung Sang, Jaekyeong Song, Hang-Yeon Weon

**Affiliations:** 1 Agricultural Microbiology Division, National Institute of Agricultural Sciences, Rural Development Administration (RDA), Wanju, 55365 South Korea; 2 Research Institute of Climate Change and Agriculture, National Institute of Horticultural & Herbal Science, RDA, Jeju, 63240 South Korea

**Keywords:** Microbial ecology, Microbial ecology, Microbiome, Microbiome

## Abstract

Plant microbiota is a key determinant of plant health and productivity. The composition and structure of plant microbiota varies according to plant tissue and compartment, which are specific habitats for microbial colonization. To investigate the structural composition of the microbiome associated with tomato roots under natural systems, we characterized the bacterial, archaeal, and fungal communities of three belowground compartments (rhizosphere, endosphere, and bulk soil) of tomato plants collected from 23 greenhouses in 7 geographic locations of South Korea. The microbial diversity and structure varied by rhizocompartment, with the most distinctive community features found in the endosphere. The bacterial and fungal communities in the bulk soil and rhizosphere were correlated with soil physicochemical properties, such as pH, electrical conductivity, and exchangeable cation levels, while this trend was not evident in the endosphere samples. A small number of core bacterial operational taxonomic units (OTUs) present in all samples from the rhizosphere and endosphere represented more than 60% of the total relative abundance. Among these core microbes, OTUs belonging to the genera *Acidovorax*, *Enterobacter*, *Pseudomonas*, *Rhizobium*, *Streptomyces*, and *Variovorax*, members of which are known to have beneficial effects on plant growth, were more relatively abundant in the endosphere samples. A co-occurrence network analysis indicated that the microbial community in the rhizosphere had a larger and more complex network than those in the bulk soil and endosphere. The analysis also identified keystone taxa that might play important roles in microbe-microbe interactions in the community. Additionally, profiling of predicted gene functions identified many genes associated with membrane transport in the endospheric and rhizospheric communities. Overall, the data presented here provide preliminary insight into bacterial, archaeal, and fungal phylogeny, functionality, and interactions in the rhizocompartments of tomato roots under real-world environments.

## Introduction

Microorganisms and plants have developed symbiotic relationships to adapt to various environmental changes. Microorganisms promote plant growth by enhancing nutrient bioavailability, suppressing plant pathogens, and increasing plant tolerance to abiotic stress factors such as drought and salinity^1,2^. In return, plants provide carbon sources, including a wide variety of sugars, amino acids, and secondary metabolites, via root exudates and tissue debris^3,4^. Understanding the intricate relationship between microbiota and host plants will improve our ability to harness these activities for increased crop productivity.

The belowground environment of plants can be classified into bulk soil (the soil that is not affected by plant roots), rhizosphere (the soil region influenced by plant roots), and endosphere (plant internal tissue). Soil is a reservoir with high microbial diversity. Distinct rhizosphere communities are shaped by microorganisms that are attracted to rhizodeposit-diffused nutrients from plant roots^5^. A subset of rhizospheric microorganisms penetrates into the plant roots and colonizes the endosphere depending on the plant’s innate immune system^5,6^. Each rhizocompartment thus harbours a microbial community with a distinct composition and structure, which can be further influenced by host-controlled mechanisms^5,7,8^. Distinct features of microbial communities between the rhizosphere and the endosphere have been observed in various types of plants, including *Arabidopsis*^8,9^, poplar^7,10^, rice^11^, agave^12^, cacti^13^, and halophytes^14^.

Microbial communities are influenced by various abiotic and biotic factors. Previous greenhouse and field studies have shown that soil type, plant genotype, plant developmental stage, and cultivation practice affect the composition and structure of microbial communities^8,9,11,15–17^. Among these factors, soil type is the strongest determinant of microbial community variation^8,9,11^, by virtue of its close association with the physicochemical properties that exert considerable influence on soil microbial diversity and biogeography^18^. In particular, pH is known to be the most critical soil characteristic affecting the composition and structure of bacterial and fungal communities across different continents^18–22^. Although the relationship between soil characteristics and soil microbial communities has been widely studied, the effect of soil characteristics on the composition and diversity of microbial communities associated with plants is largely unknown.

A wide variety of microorganisms have been detected in soil, including bacteria, archaea, fungi, viruses, and other microbial eukaryotes^23^. Despite this microbial diversity, microbiome studies have focused mainly on bacteria that are phylogenetically well- characterized. Fungi and archaea are closely associated with plant health and have significant biodiversity in the rhizosphere, with concentrations ranging from 10^5^ to 10^6^ and 10^7^ to 10^8^ per g of rhizospheric soil, respectively^24^. Some fungal strains of the genera *Trichoderma* and *Fusarium* exhibit biocontrol activity^25,26^, and mycorrhizal fungi are well-known providers of mineral nutrition for plants^27^. Ammonium-oxidizing archaea are more abundant in the rhizosphere than in bulk soil^28^. Moreover, interactions among the three kingdoms, such as interactions between bacteria and fungi, are known to promote plant growth^29^. To obtain a comprehensive understanding of microbial communities, it is necessary to integrate studies on the three kingdoms and focus on the interactions within the microbial network. The highly connected taxa within the microbial network are considered to be keystone taxa that play an important role in the structure and function of microbial communities^30^.

The tomato is one of the most widely grown vegetables, with an annual production of more than 170 million tons worldwide (http://faostat.fao.org). This plant has been well-studied as a plant model in genetics and genomics, but research on the tomato microbiome remains in its infancy^31^. Research on the bacterial and fungal communities on the surfaces of tomato plant organs (leaves, stems, roots, flowers, and fruits)^32^ and the leaf endosphere has been going on since 2013^31^. The effect of supplementing soil with organic and synthetic fertilizers on bacterial communities in the rhizosphere and phyllosphere of the tomato plants was examined through the presence and absence of the oomycete plant pathogen, *Phytophthora parasitica*^33,34^. The bacterial communities and functions of endophytes affected by nematode pathogenesis were also analysed^35^. To the best of our knowledge, no studies have been published on the characterization of the phylogenetic and functional diversity of the three kingdoms and their interactions within the different compartments of tomato roots under natural systems.

In the present study, we conducted a preliminary investigation of microbial communities in the rhizosphere, endosphere, and bulk soil samples of tomatoes cultivated in 23 geographically different greenhouses. The three kingdoms, namely, bacteria, fungi, and archaea, were examined using the Illumina MiSeq platform with three pairs of specific primers targeting the 16S rRNA genes and the internal transcribed spacer (ITS) region. Distinct microbial communities were identified according to tomato rhizocompartments, regardless of differences in soil characteristics, and examined for species diversity, effects of edaphic factors, representative taxa, microbial network topology, and predictive functional gene profiles using various bioinformatics tools.

## Results

### Microbial richness and diversity of the different rhizocompartments of tomatoes

**Figure 1:**
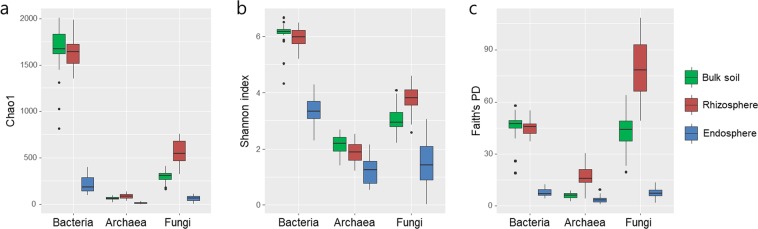
Richness and diversity of microbial communities within rhizocompartments. Richness was estimated by the Chao1 (**a**), and diversity was represented by Shannon’s index (**b**) and Faith’s PD (**c**). Sequence reads of bulk soil and rhizosphere samples were rarefied to 20,000 and those of endosphere samples to 4,000. The box plots exhibit the range of variation in the median values (black lines in the middle), and the dots depict the outliers.

To investigate the features of the microbial communities associated with different rhizocompartments of tomatoes, tomato plants were collected from 23 greenhouses located in 7 different geographic locations across South Korea (Supplementary Fig. S1 and Supplementary Table S1). Roots of collected tomato plants were separated according to bulk soil, rhizosphere, and endosphere^8,9^. A total of 69 samples (23 greenhouses × 3 compartments) were used for Illumina MiSeq sequencing with bacteria-, fungi-, and archaea-specific primers. The number of high-quality sequences generated from high-throughput sequencing was 7,985,514 bacterial 16S rRNA gene sequences, 4,637,772 archaeal 16S rRNA gene sequences, and 6,979,207 fungal ITS region sequences. After removing operational taxonomic units (OTUs) assigned to non-target kingdoms or chloroplasts, 4,387,870 bacterial, 4,184,774 archaeal, and 2,582,195 fungal sequences were clustered into 3,511 bacterial, 678 archaeal, and 4,608 fungal OTUs. Although the number of generated sequences and OTUs was different among the kingdoms and rhizocompartments, the rarefaction curves showing the observed number of OTUs of those samples were sufficiently saturated (Supplementary Fig. S2). Bacterial OTU richness was the highest and archaeal OTU richness was the lowest among the three kingdoms. After eliminating sequences assigned to the chloroplasts of plants, the number of sequences and OTUs of the endosphere samples were exceedingly low in comparison to those of the bulk soil and rhizosphere samples. The OTU datasets of endosphere samples were rarefied to 4,000 reads while those of bulk soil and rhizosphere were rarefied to 20,000 reads, both of which are close to the depth of saturation with more than 98% coverage (Supplementary Table S2). To compare significant differences in species diversity between samples, α-diversity was represented by the Chao1 richness estimate and Shannon’s diversity index based on the rarefied OTU datasets (Fig. 1 and Supplementary Table S2). The communities belonging to the three kingdoms, particularly bacteria and archaea, had similar levels of α-diversity in the bulk soil and rhizosphere, with the highest values observed for bacteria, followed by fungi and archaea. Of the rhizocompartments, the microbial communities of the endosphere exhibited the lowest Chao1 richness estimate and Shannon’s diversity index in comparison to those of the bulk soil and rhizosphere across all three kingdoms. Similarly, the Faith’s phylogenetic diversity (Faith’s PD) was found to be the lowest in the endosphere compared to the bulk soil and rhizosphere (Fig. 1c).

### Differences in microbial community structure between rhizocompartments

**Figure 2:**
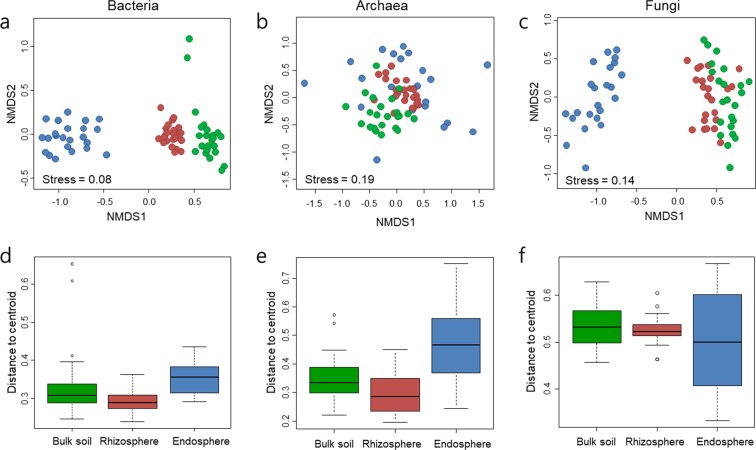
Similarity and variation among microbial communities within rhizocompartments. Non-metric multidimensional scaling (NMDS) plots analysed with Bray-Curtis distance matrices of bacterial (**a**), archaeal (**b**), and fungal (**c**) communities after normalizing OTU dataset with Hellinger transformation. The colours of the dots denote the compartments of the samples: bulk soil (green), rhizosphere (red), and endosphere (blue). The box plots represent the range of distances from the centroid based on the Bray-Curtis distance matrices of bacterial (**d**), archaeal (**e**), and fungal (**f**) compositions. The black lines in the box plots denote median values, and the dots depict outliers.

We examined the similarities in the microbial communities between samples via a non-metric multidimensional scaling (NMDS) biplot based on Bray-Curtis dissimilarity (Fig. 2a–c) and Principal Coordinates Analysis (PCoA) of weighted UniFrac distances incorporating phylogenetic relatedness (Supplementary Fig. S3). Despite the different soil types that the tomatoes were grown in, the microbial communities were separated by rhizocompartments. In particular, the bacterial communities were clearly separated by three compartments, and those in the endosphere were found to retain the most distinguishable bacterial communities. The archaeal communities were separated between the bulk soil and rhizosphere, while the endosphere exhibited a community signature that was distinct from those of the other two compartments. The fungal communities exhibited similar community structures as the bacterial communities, with the endosphere harbouring the most distinct communities. However, the fungal communities were not differentiated between the bulk soil and rhizosphere. The ordination results were further supported by a non-parametric analysis of similarities (ANOSIM) and permutational multivariate analysis of variance (PERMANOVA) based on Bray-Curtis dissimilarity. ANOSIM revealed significant separation of the bacterial (R = 0.865, *P* < 0.001) and fungal (R = 0.756, *P* < 0.001) communities by rhizocompartment, while the archaeal communities exhibited low values (R = 0.308, *P* < 0.001). PERMANOVA also demonstrated that the bacterial (R^2^ = 0.477, *P* < 0.001), fungal (R^2^ = 0.260, *P* < 0.001), and archaeal (R^2^ = 0.179, *P* < 0.001) communities were significantly differentiated by rhizocompartment.

Next, the homogeneity of communities within the same compartment was examined by measuring the distance between the centroid and each sample of the group. The bacterial, archaeal, and fungal communities of the rhizosphere exhibited the lowest dispersion, while those of the endosphere exhibited higher dispersion than those of the other compartments (Fig. 2d–f). Taken together, the results show that the endosphere harbours much fewer species but exhibits greater variation in microbial community structure than the rhizosphere and bulk soil.

### Influence of soil characteristics on the microbial communities of tomato roots

Physicochemical analyses of the collected bulk soils provided a wide range of values across the samples (Supplementary Table S3). The soil pH and electrical conductivity (EC) values varied from 4.8 to 7.8 and 0.1 to 4.0 dS/m, respectively. The organic matter (OM) content ranged from 12.1 to 72.6 g/kg. The total nitrogen (TN) content varied from 0.07 to 0.55%. The available phosphate content varied from 98.9 to 1319.0 mg/kg. The exchangeable cations, such as K^+^, Ca^2+^, Mg^2+^, and Na^+^, varied from 0.1 to 17.6 cmol_c_/kg.

We investigated whether the microbial communities inhabiting the bulk soil, rhizosphere, and endosphere are influenced by edaphic factors using the Mantel test and canonical correspondence analysis (CCA). Overall, the bacterial and fungal communities within the bulk soil and rhizosphere exhibited significant correlations with the edaphic factors measured in this study (Table 1 and Supplementary Fig. S4). Interestingly, the microbial communities within the endosphere had no significant correlation with any edaphic factors. Among the three kingdoms, relatively few edaphic factors were associated with archaeal communities. The communities of bacteria and fungi in the rhizosphere were more greatly influenced by the edaphic factors than those in the bulk soil. The Mantel test showed that soil pH, TN, K^+^, Ca^2+^, and Mg^2+^ levels were significantly correlated with the bacterial communities in the bulk soil. In addition to these edaphic factors, the bacterial communities in the rhizosphere exhibited a significant relationship with EC, OM, and Na^+^ levels. The fungal communities within the bulk soil had significant relationships with pH, EC, Mg^2+^, and Na^+^ levels, and those in the rhizosphere were additionally correlated with TN and K^+^ levels. The archaeal communities in the bulk soil were correlated with pH, TN, and Mg^2+^ levels, while the rhizospheric archaeal communities were correlated with only Mg^2+^ levels. Similar to the Mantel test, the results of CCA showed that pH was the main factor that shaped the bacterial and fungal communities in the bulk soil and rhizosphere (Supplementary Fig. S4).

**Table 1:** Correlations between microbial community and environmental variables using the Mantel test.

Environmental variables	Bacteria			Archaea			Fungi		
	B	R	E	B	R	E	B	R	E
pH	0.603**	0.398**	0.097	0.417**	0.208	−0.043	0.270*	0.279*	0.080
EC	0.283	0.281*	0.131	0.195	0.197	−0.039	0.356**	0.391**	−0.128
OM	0.238	0.259*	0.203	0.165	0.001	−0.122	0.028	0.099	−0.091
TN	0.483**	0.343**	0.125	0.341*	0.148	−0.112	0.154	0.203*	−0.085
Avail. P_2_O_5_	0.241	0.066	−0.054	0.151	0.012	−0.249	0.063	0.078	0.008
K^+^	0.334*	0.247*	0.144	0.213	0.001	−0.1	0.201	0.241*	0.175
Ca^2+^	0.353*	0.405**	0.218	0.259	0.235	−0.083	0.159	0.192	−0.167
Mg^2+^	0.508*	0.464**	0.205	0.399*	0.348*	−0.049	0.295**	0.365**	−0.117
Na^+^	0.254	0.310**	0.039	0.178	0.154	−0.045	0.269*	0.340**	−0.088

Bray-Curtis dissimilarity was calculated for the microbial community composition, and Euclidean distance was calculated for the environmental variables. The numbers indicate the Mantel statistic (r) value, with significance as indicated (**p < 0.001, *p < 0.01). Abbreviations: B, bulk soil; R, rhizosphere; E, endosphere; EC, electrical conductivity; OM, organic matter; TN, total nitrogen; Avail., available.

Furthermore, forward selection based on redundancy analysis (RDA) was implemented to quantify the relative contributions of the selected soil physicochemical properties to the structures of the microbial communities^36^. Microbial communities in the bulk soil and rhizosphere were highly influenced by soil edaphic factors, but this trend was not observed in the endosphere, as demonstrated by the Mantel test. Specifically, a combination of the selected soil parameters, including pH, EC, K^+^, and Ca^2+^, explained 27.9% of the bacterial community variance in the bulk soil, and K^+^, OM, pH, and Na^+^ explained 21.1% of the community variance in the rhizosphere (Supplementary Table S4). A combination of pH and Mg^2+^ explained 18.4% of the archaeal community variance in the bulk soil, and pH alone explained 12.0% of the community variance in the rhizosphere. In the fungal communities, a combination of EC, Na^+^, and Ca^2+^ explained 16.3% of the community variance in the bulk soil, while Na^+^ and pH explained only 7.8% of the community variance in the rhizosphere. However, the selected soil parameters of the bacterial, archaeal, and fungal communities in the endosphere either did not explain the microbial community variance (no parameter explained the variance in the archaeal communities) or the explanation provided was unsatisfactory (cumulative variance of 7.1% and 7.6% in bacterial and fungal communities, respectively).

### Microbial taxonomic distribution in different rhizocompartments of tomatoes

**Figure 3:**
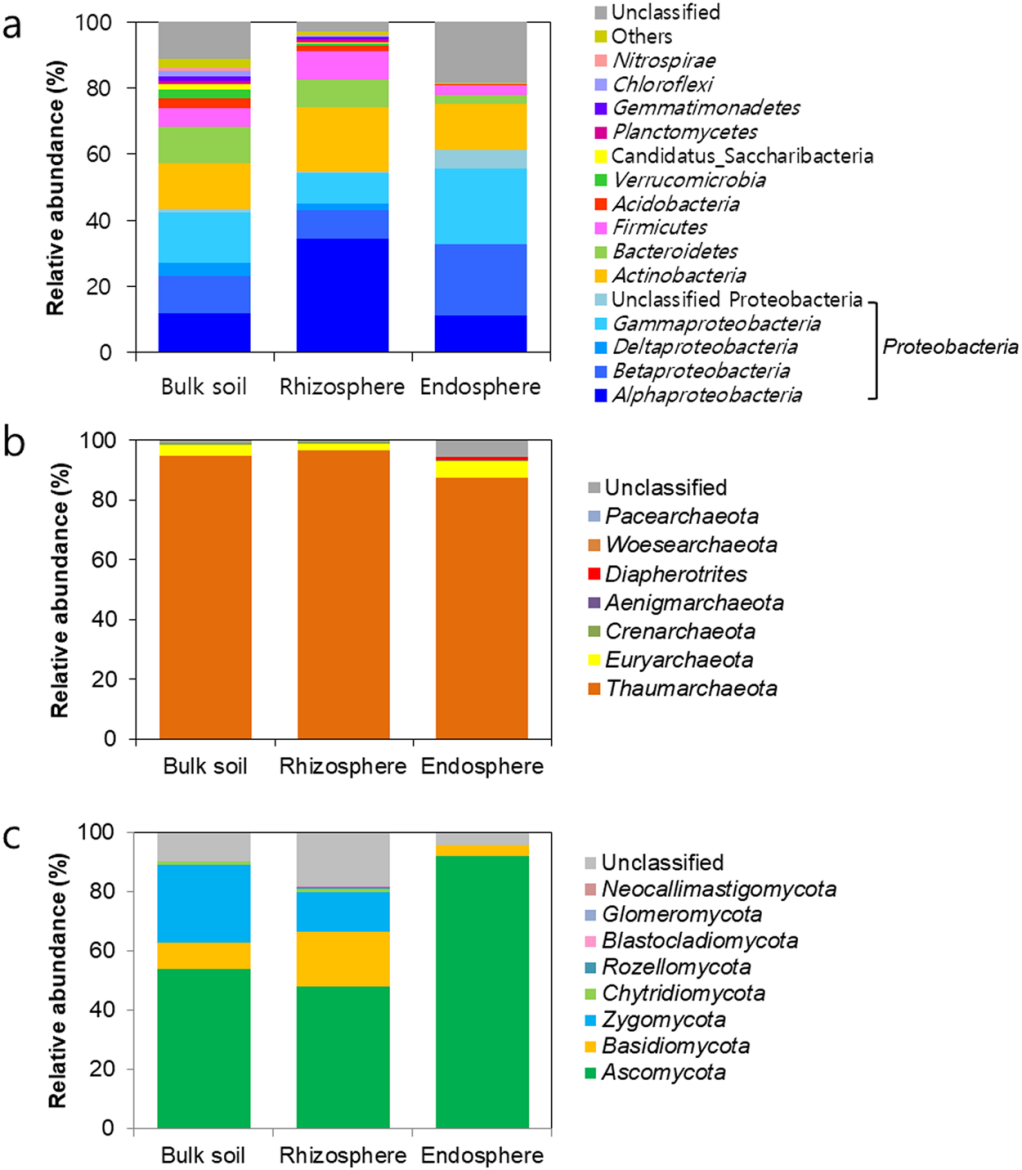
Comparison of taxonomic distributions between rhizocompartments. The average relative abundances of bacterial (**a**), archaeal (**b**), and fungal (**c**) phyla are represented according to rhizocompartment. The phyla with relative abundances less than 1% were classified into “Others”.

We detected a total of 25 bacterial, 7 archaeal, and 8 fungal phyla from the dataset, and the taxonomic distributions were presented according to rhizocompartment with average relative abundances (Fig. 3). The communities of the three kingdoms exhibited distinct taxonomic distribution patterns between the bulk soil, rhizosphere, and endosphere at the phylum level. Among the bacterial communities, the most dominant phylum was *Proteobacteria*, with relative abundances of 43.2 ± 6.2%, 54.7 ± 6.5%, and 61.2 ± 14.7% in the bulk soil, rhizosphere, and endosphere, respectively. At the class level, the relative abundances of *Alphaproteobacteria* (11.8 ± 2.8%), *Betaproteobacteria* (11.2 ± 5.5%), and *Gammaproteobacteria* (15.0 ± 10.4%) were similar in the bulk soil. In the rhizosphere, the relative abundance of *Alphaproteobacteria* (34.4 ± 9.3%) was the highest, while the relative abundances of *Betaproteobacteria* (21.3 ± 12.1%) and *Gammaproteobacteria* (21.8 ± 11.1%) were higher than that of *Alphaproteobacteria* (11.1 ± 6.6%) in the endosphere. *Actinobacteria* was the second most abundant phylum, with relative abundances of 14.1 ± 6.2%, 19.5 ± 4.4%, and 13.8 ± 14.9% in the bulk soil, rhizosphere, and endosphere, respectively. The relative abundances of *Bacteroidetes* (2.9 ± 1.6%) and *Firmicutes* (2.9 ± 3.5%) in the endosphere were 2 to 3 times lower than those in the bulk soil and rhizosphere. Unlike the bacterial communities, the archaeal communities were predominated by a few dominant phyla. The most dominant archaeal phylum was *Thaumarchaeota*, with relative abundances of 94.7 ± 4.8%, 96.7 ± 4.5%, and 87.4 ± 22.0% in the bulk soil, rhizosphere, and endosphere, respectively. *Euryarchaeota* was the second most abundant archaeal phylum, with relative abundances of 3.6 ± 4.1%, 2.2 ± 2.7%, and 5.5 ± 12.2% in the bulk soil, rhizosphere, and endosphere, respectively. The fungal communities were also dominated by a few dominant phyla. *Ascomycota* was the most abundant phylum, with relative abundances of 54.1 ± 18.8%, 54.5 ± 15.5% and 93.9 ± 13.9% in the bulk soil, rhizosphere, and endosphere, respectively. While *Ascomycota* exclusively occupied the endophytic fungal communities, the bulk soil and rhizosphere harboured significant proportions of *Basidiomycota* (8.7 ± 9.3%, 18.5 ± 16.7%, and 3.5 ± 12.3% in the bulk soil, rhizosphere, and endosphere, respectively) and *Zygomycota* (26.4 ± 20.8%, 13.2 ± 9.9%, and 0.2 ± 0.2% in the bulk soil, rhizosphere, and endosphere, respectively) in addition to *Ascomycota*.

### Identification of representative OTUs in each rhizocompartment

We took a closer look at the individual OTUs abundant in each rhizocompartment of the tomato roots. To identify the taxa that were significantly associated with particular ecological niches, a species indicator analysis was performed. Six OTUs, 14 OTUs, and 19 OTUs with high indicator values (>0.5) and dominance (>1% average relative abundance) were identified in the bulk soil, rhizosphere, and endosphere, respectively (Table 2). Bacterial OTUs were found mainly as indicator taxa across all compartments (23 out of 39 OTUs) and were mostly affiliated with *Actinobacteria* and *Proteobacteria*. The fungal indicator taxa in the three compartments (15 out of 39 OTUs) were affiliated with *Ascomycota*. Only one archaeal OTU, belonging to *Thaumarchaeota*, was detected in the bulk soil; however, no significant archaeal indicator taxa were identified in the rhizosphere and endosphere. At the genus level, the indicator OTUs of the rhizosphere were assigned to *Microbacterium*, *Arthrobacter*, *Sphingobium*, *Sphingomonas*, *Afipia*, *Leifsonia*, *Luteimonas*, *Hyphodiscus*, *Aspergillus*, *Trichoderma*, *Chrysosporium*, and *Oidiodendron*, and those of the endosphere were affiliated with *Enterobacter*, *Acidovorax*, *Variovorax*, *Pseudomonas*, *Rhizobium*, *Streptomyces*, *Alternaria*, and *Colletotrichum*. Linear discriminant analysis (LDA) Effect Size (LEfSe) is a statistical method that determines the features (organisms, genes, and functions that show a significant difference between groups. We conducted an LEfSe analysis to identify specific taxa in different rhizocompartments that showed an LDA score > 3.5 and *p* < 0.05. Eight, 6, and 13 OTUs were identified in the bulk soil, rhizosphere, and endosphere, respectively (Supplementary Fig. S5). Most of the OTUs identified in the endosphere were shared with the OTUs identified by the indicator species analysis.

**Table 2:** Indicator taxa.

Compartment	OTU	Phylum/Class	Order/Family/Genus	Indicator value	Relative abundance (%)		
					B	R	E
Bulk	B_OTU13	*Betaproteobacteria*	*Burkholderiales*	0.778	1.38	0.12	0.02
	B_OTU43	*Gammaproteobacteria*	Unclassified	0.773	1.13	0.14	0.02
	B_OTU48	*Betaproteobacteria*	*Burkholderiales*	0.670	1.64	0.11	0.02
	B_OTU28	*Betaproteobacteria*	*Burkholderiales*	0.576	2.13	0.05	0.01
	F_OTU28	*Ascomycota*	*Pyronemataceae*	0.574	5.27	0.12	0
	A_OTU4	*Thaumarchaeota*	*Nitrosopumilus*	0.533	15.51	1.25	3.93
Rhizosphere	B_OTU106	*Alphaproteobacteria*	*Phyllobacteriaceae*	0.685	0.30	1.28	0.12
	B_OTU7	*Actinobacteria*	*Microbacterium*	0.676	0.24	2.42	0.76
	B_OTU4	*Actinobacteria*	*Arthrobacter*	0.667	0.41	3.18	0.16
	B_OTU2	*Alphaproteobacteria*	*Sphingobium*	0.664	0.16	10.68	2.43
	B_OTU50	*Alphaproteobacteria*	*Sphingomonas*	0.653	1.51	3.26	0.10
	B_OTU77	*Alphaproteobacteria*	*Afipia*	0.643	0.11	1.12	0.28
	B_OTU16	*Actinobacteria*	*Leifsonia*	0.641	0.19	1.11	0.42
	F_OTU799	*Ascomycota*	*Hyphodiscus*	0.633	0	0.01	0
	B_OTU35	*Gammaproteobacteria*	*Luteimonas*	0.603	0.66	2.27	1.14
	F_OTU364	*Ascomycota*	*Aspergillus*	0.574	0	0.03	0
	B_OTU15	*Betaproteobacteria*	Unclassified	0.547	0.41	1.01	0.03
	F_OTU609	*Ascomycota*	*Trichoderma*	0.535	0.01	0.03	0.0002
	F_OTU31	*Ascomycota*	*Chrysosporium*	0.525	0.14	1.46	0.01
	F_OTU158	*Ascomycota*	*Oidiodendron*	0.520	0	0.11	0.0003
Endosphere	B_OTU18	*Gammaproteobacteria*	*Enterobacter*	0.798	0.03	0.21	6.38
	B_OTU26	*Betaproteobacteria*	*Acidovorax*	0.767	0.01	0.16	2.84
	B_OTU33	Unclassified	Unclassified	0.750	0	0.002	4.22
	B_OTU19	Unclassified	Unclassified	0.735	0	0.002	11.42
	B_OTU45	*Proteobacteria*	Unclassified	0.725	0	0.0007	5.56
	B_OTU6	*Betaproteobacteria*	*Variovorax*	0.619	0.13	1.37	10.08
	B_OTU3	*Gammaproteobacteria*	*Pseudomonas*	0.580	0.43	1.19	10.37
	F_OTU42	*Ascomycota*	*Alternaria*	0.580	0	0.008	3.12
	F_OTU1464	*Ascomycota*	*Alternaria*	0.570	0	0.01	1.89
	F_OTU53	*Ascomycota*	*Alternaria*	0.569	0	0.008	2.14
	B_OTU17	*Alphaproteobacteria*	*Rhizobium*	0.568	0.01	0.52	1.67
	F_OTU121	*Ascomycota*	*Alternaria*	0.567	0	0.007	1.17
	F_OTU27	*Ascomycota*	Unclassified	0.561	0	0.02	5.92
	B_OTU23	*Actinobacteria*	*Streptomyces*	0.561	0.35	0.72	2.86
	F_OTU196	*Ascomycota*	*Alternaria*	0.546	0	0.001	0.69
	F_OTU477	*Ascomycota*	*Alternaria*	0.540	0	0.002	0.23
	F_OTU2	*Ascomycota*	*Colletotrichum*	0.523	0.02	1.26	25.91
	F_OTU295	*Ascomycota*	*Alternaria*	0.522	0	0.003	0.66
	B_OTU199	*Gammaproteobacteria*	*Pseudomonas*	0.512	0.12	0.11	1.27

The data table shows the results for the indicator species analysis with point biserial correlation. The OTUs with high indicator values (>0.5) and high abundances (>1% relative abundance) are listed. Abbreviations: B_OTU, bacterial OTU; F_OTU, fungal OTU; A_OTU, archaeal OTU; B, bulk soil; R, rhizosphere; E, endosphere. The significance level is p < 0.001.

**Figure 4:**
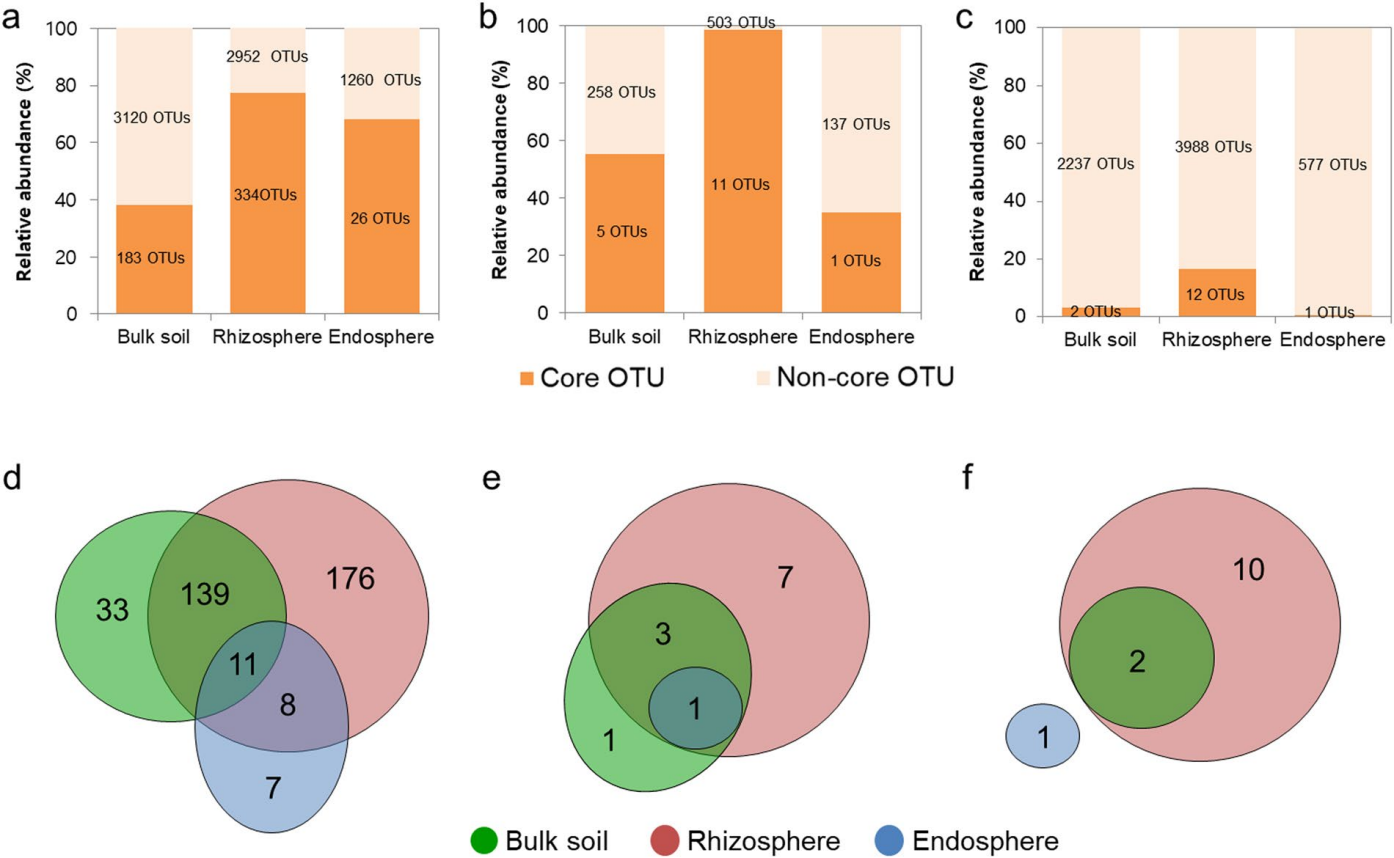
Number of core OTUs in the rhizocompartments. The bar graphs represent the relative abundances of core OTUs (orange) in the bacterial (**a**), archaeal (**b**), and fungal (**c**) communities. The Venn diagrams represent the number of core bacterial (**d**), archaeal (**e**), and fungal (**f**) OTUs that were detected from all the samples in the bulk soil, rhizosphere, and endosphere.

To identify the persistent members of microbial communities that inhabit each rhizocompartment, we identified core OTUs that were detected in all the bulk soil, rhizosphere and endosphere samples (Fig. 4). The bacterial core microbiomes comprised 183, 334, and 26 OTUs in the bulk soil, rhizosphere, and endosphere, respectively, accounting for relative abundances of 38%, 77%, and 68%, respectively (Fig. 4a). The archaeal core microbiomes comprised 1–11 OTUs with relative abundances of 34.9–98.8% (Fig. 4b). Archaeal OTU1, which was a core OTU in all rhizocompartments, was abundant exclusively in the rhizosphere (50%), endosphere (35%), and bulk soil (18%). In contrast to bacteria and archaea, the number of fungal core OTUs was small (1–12 OTUs) despite the large total OTU number (Fig. 4c). Next, core OTUs in the rhizosphere and endosphere that might be closely associated with plants were phylogenetically classified. Nineteen bacterial core OTUs were common to both the rhizosphere and endosphere, belonging to *Pseudomonas*, *Variovorax*, *Enterobacter*, *Rhizobium*, *Shinella*, *Acidovorax*, *Rhizobium*, *Propionibacteriaum*, *Sphingobium*, *Luteimonas*, *Rhodococcus*, and *Streptomyces* (Fig. 4d and Supplementary Data S1). An archaeal core OTU that was common to all the compartments was affiliated with *Nitrososphaera*, and in addition, the rhizospheric core OTUs were assigned to *Methanobacterium* and *Methanosarcina* (Fig. 4e and Supplementary Data S1). The fungal core OTUs in the bulk soil and rhizosphere were assigned to *Cladosporium*, *Fusarium*, *Chrysosporium*, *Trichocladium*, *Monographella*, and *Gibellulopsis* (Fig. 4f and Supplementary Data S1). The one fungal core OTU in the endosphere belonged to *Alternaria*.

### Complexity of the microbial network among rhizocompartments of tomatoes

Potential interactions between microbial taxa in each rhizocompartment were investigated using a random matrix theory (RMT)-based network analysis by combining bacterial, fungal, and archaeal OTUs. For network construction, we used 1,619, 1,633, and 160 OTUs for bulk soil, rhizosphere, and endosphere, respectively, which were detected in more than half of the samples as recommended by the network analyses pipeline (Table 3). The network connectivity in all compartments was well fitted by the power law, with an R^2^ value greater than 0.7, indicating scale-free properties. The values for the average clustering coefficient (avgCC) and average path distance (GD) of the empirical networks were higher than those of the random networks, indicating the small-world behaviour of the networks. The number of nodes in the rhizospheric network was lower, but the average degree (avgK) was higher, than that in the bulk soil network. This finding suggested that microbial communities in the rhizosphere formed more complex networks than those in bulk soil (Table 3 and Supplementary Fig. S6). Compared to the rhizosphere, the endosphere exhibited low and simple network connectivity, as indicated by the small number of nodes and low avgK value.

**Table 3:** Topological properties of the networks of microbial communities in the bulk soil, rhizosphere, and endosphere.

Network Indices	Bulk soil	Rhizosphere	Endosphere
**Empirical network**			
Commonly present OTU No. (n > 12)	1619	1633	160
Total nodes	652	564	117
Total links	1026	1335	183
Similarity threshold (St)	0.83	0.86	0.62
R square of power-law	0.875	0.746	0.745
Average degree (avgK)	3.147	4.734	3.128
Average clustering coefficient (avgCC)	0.112	0.142	0.202
Average path distance (GD)	7.847	6.968	6.931
Harmonic geodesic distance (HD)	5.975	5.639	4.973
Density (D)	0.005	0.008	0.027
Number of modules	84	56	10
Modularity	0.787	0.856	0.752
**Random network**			
Average clustering coefficient (avgCC)	0.008	0.013	0.03
Average path distance (GD)	4.83	4.018	4.074

Next, the topological roles of the taxa in the network were classified into the following four categories based on the values of within-module connectivity (Zi) and among-module connectivity (Pi): peripherals, few interactions with other nodes (Zi < 2.5 and Pi < 0.62); connectors, many links with other modules (Zi < 2.5 and Pi > 0.62); module hubs, many interactions within the module (Zi > 2.5 and Pi < 0.62); and network hubs, many interactions within and among modules (Zi > 2.5 and Pi > 0.62) (Supplementary Fig. S7)^30,37^. Most of the nodes (99%, 98%, and 88% in bulk soil, rhizosphere, and endosphere, respectively) were categorized as peripherals with low connectivity. None of the nodes fell into the network hub category. Notably, 9 module hubs and 16 connectors were identified from the dataset and are considered to be keystone taxa, with potential roles in shaping distinct microbial communities in the bulk soil, rhizosphere, and endosphere (Supplementary Table S5). Among the 9 module hubs, 3 module hubs in the bulk soil network were assigned to *Gemmatimonadetes*, *Firmicutes*, and *Proteobacteria*; 5 module hubs in the rhizospheric network were assigned to *Actinobacteria*, *Bacteroidetes*, and *Alphaproteobacteria*; and one module hub in the endospheric network was assigned to *Firmicutes*. Of the 15 connectors, 12 connectors in the bulk soil network were assigned to *Planctomycetes*, *Actinobacteria*, *Proteobacteria*, *Elusimicrobia*, and *Firmicutes*; 3 connectors in the rhizospheric network were assigned to *Proteobacteria* and *Ignavibacteriae*; and there was no connector identified in the endosphere.

### Metagenome prediction profiles of each rhizocompartment of tomatoes

**Figure 5:**
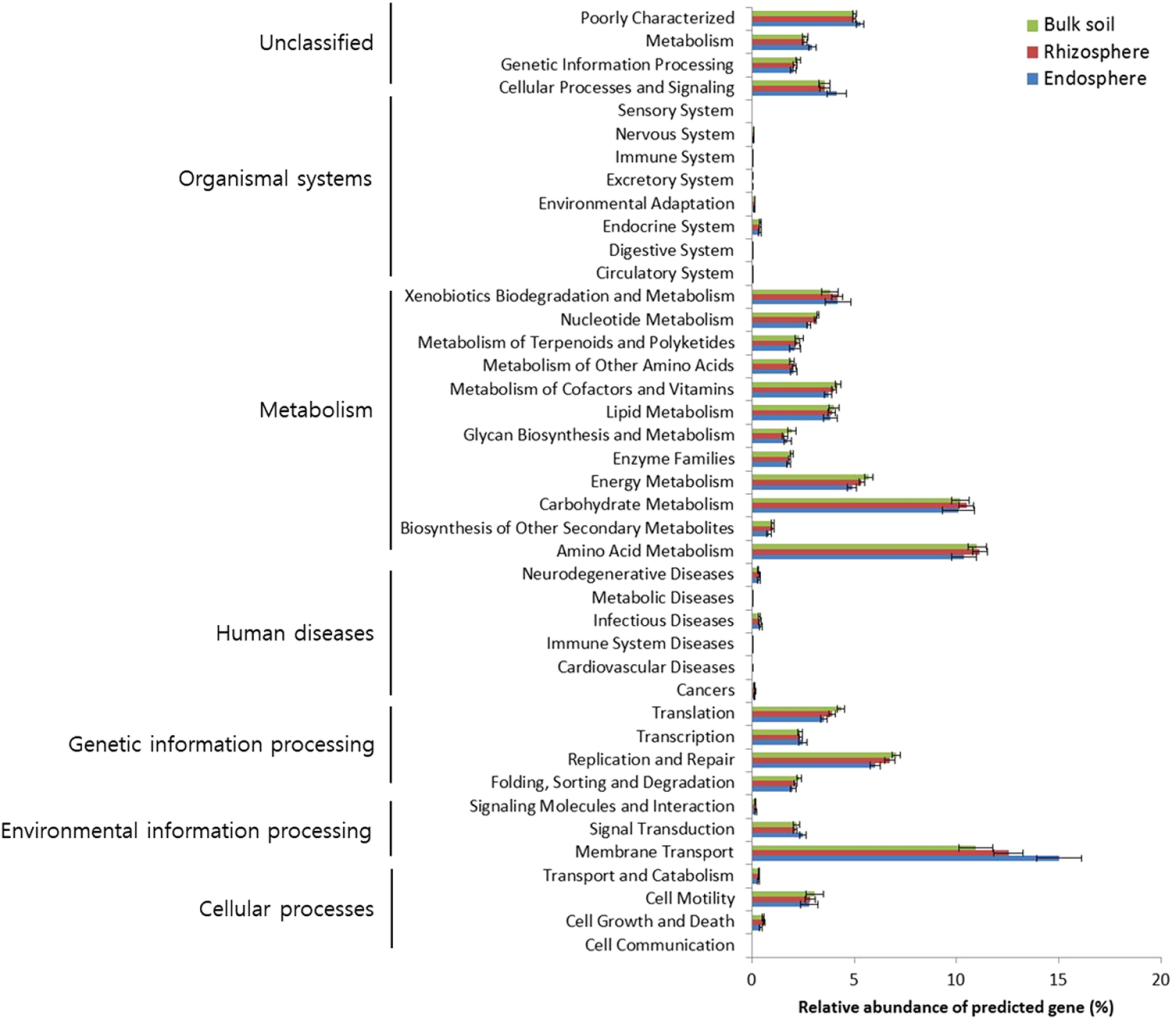
Gene profiles of the bacterial communities in the bulk soil, rhizosphere, and endosphere based on functional categories (KEGG database, level 2) predicted using PICRUSt.

Functional gene profiles of each compartment were predicted using the PICRUSt program with the bacterial 16S rRNA gene sequence dataset. Based on the Kyoto Encyclopedia of Genes and Genomes (KEGG) pathway hierarchy level 2, 41 KEGG orthology (KO) groups were identified (Fig. 5). Functional genes belonging to carbohydrate metabolism, amino acid metabolism, and membrane transport were markedly abundant in the dataset. Among these genes, the relative abundances of genes predicted to be associated with membrane transport exhibited high variability among rhizocompartments, with the highest abundance observed in the endosphere followed by the rhizosphere and bulk soil. This finding was further supported by a higher abundance of genes associated with the sublevel of membrane transport (ABC transporter, secretion system, transporters, and phosphotransferase system) at the third hierarchical level in the endosphere than in the other two compartments (Supplementary Fig. S8).

## Discussion

We collected tomato plants cultivated in 23 greenhouses in 7 different geographic locations across South Korea and performed in-depth characterization of the microbial communities harboured in different rhizocompartments using the Illumina MiSeq platform. Among the three rhizocompartments, both taxonomic and phylogenetic diversity of the endosphere was considerably lower than that of the rhizosphere and bulk soil (Fig. 1). The microbial alpha-diversity, estimated by the Chao1 richness index and the Shannon’s diversity index, was highest among bacterial communities, followed by fungal and archaeal communities. Despite the differences in soil characteristics, analysis of the microbial community structure revealed a clear differentiation between bulk soil, rhizosphere, and endosphere, as demonstrated by the beta-diversity analysis (NMDS, ANOSIM, and PERMANOVA based on Bray-Curtis dissimilarity and PCoA based on weighted UniFrac distances) (Fig. 2 and Supplementary Fig. S3). Soil microorganisms attracted to root exudates are reassembled in the rhizosphere. A subset of rhizospheric microorganisms that have endophytic competence and interact with the plant innate immune system colonize the endosphere^5,6,10,38^. Intriguingly, the ordination results analysed by NMDS showed that the variation in the microbial community structure of the endosphere was considerably higher than that of the bulk soil and rhizosphere (Fig. 2). This finding was consistent with those of previous microbiome studies of poplar trees, which showed high structural variation among microbial communities within the endosphere in comparison to those in the rhizosphere^7,10^. The high variance in microbial communities in the endosphere might be dependent on the plant innate immune system, while rhizospheric microbial communities are less influenced by host-dependent selection^10^. Taken together, the results show that microbial communities of a tomato’s endosphere are characterized by a low species richness and diversity and a high vulnerability to plant responses, resulting in a high degree of variation in the microbial community structure.

We examined soil samples that had different physiochemical properties to evaluate whether edaphic factors affect the composition and diversity of microbial communities, not only in the soil but also in the rhizosphere and endosphere. The Mantel test and CCA results showed that soil physicochemical properties were correlated with microbial communities in the rhizosphere and bulk soil but not in the endosphere (Table 1 and Supplementary Fig. S4). While the microbial communities in the rhizosphere could be affected by edaphic factors of the surrounding soil, the endosphere is isolated from the soil by the plant’s epidermal cells and may be influenced by the plant’s innate immune system rather than by soil properties. Thus, edaphic factors are among the determinants of the structures of microbial assemblages found in the bulk soil and rhizosphere, but not in the endosphere. Among the edaphic factors measured, pH was a major factor influencing bacterial and fungal communities in both the bulk soil and rhizosphere, similar to what has been previously reported^18–22,39,40^. Archaeal communities were also influenced by soil pH in the bulk soil and rhizosphere. Moreover, one of the cations, namely Mg^2+^, which is an important cofactor of ATP and is involved in the regulation of metabolic reactions and energy balance of organisms^41^, was significantly correlated with the bacterial, archaeal, and fungal communities. While EC and other cations, including K^+^ and Na^+^, were correlated with bacterial and fungal communities, the archaeal communities were not affected by other soil characteristics, suggesting that archaeal communities are less influenced by environmental factors than bacterial and fungal communities.

We identified specific microbial taxa that were significantly more relatively abundant in each rhizocompartment under various environmental conditions. The indicator species and LEfSe analysis identified representative OTUs that were significantly more relatively abundant in bulk soil, rhizosphere, and endosphere (Table 2 and Supplementary Fig. S5). Bacterial OTUs that were specifically more relatively abundant in the endosphere were affiliated with *Enterobacter* (B_OTU18), *Acidovorax* (B_OTU26), *Variovorax* (B_OTU6), *Pseudomonas* (B_OTU3), *Rhizobium* (B_OTU17), and *Streptomyces* (B_OTU23). Notably, these genera have been previously reported to promote plant growth, biocontrol, and nutrient availability^42–46^. Interestingly, the other OTUs abundant in the endosphere were affiliated with the fungal genera *Alternaria* (F_OTU42, 53, 121, 196, 295, 464, and 477) and *Colletotrichum* (F_OTU2) within the phylum *Ascomycota*. Some *Alternaria* species, such as *A*. *alternata* and *A*. *solani*, are known tomato pathogens that cause leaf spots, rots, and blights^47^. Species belonging to *Colletotrichum*, such as *C*. *acutatum*, *C*. *gloeosporioides*, *C dematium*, and *C*. *coccodes*, are known causes of anthracnose in tomato plants^48^. However, no symptom of anthracnose was evident on the tomato plants used in this study. Apparently, the OTUs belonging to the two genera may be non-virulent in tomato plants but are effective in endophytic colonization. Among the genera associated with indicator OTUs in the rhizosphere, members of *Sphingobium* (B_OTU2), *Arthrobacter* (B_OTU4), and *Sphingomonas* (B_OTU50) are known to promote plant growth^5,49^. Moreover, in the rhizosphere, species of the fungal indicator taxa, which included *Aspergillus* (F_OTU364), *Trichoderma* (F_OTU4), and *Chrysosporium* (F_OTU31), have also been reported to have plant growth-promoting activity, mainly by producing plant hormones such as gibberellin and auxin^50–52^. In general, a greater number of taxa that positively affect plant growth and health were found in the rhizosphere and endosphere in comparison to bulk soil samples.

We identified the core OTUs that were present in all 23 tomato samples. Given that the core microbiome is a stable and consistent component associated with a particular habitat, these microbes are thought to play important roles in the ecosystem^53^. The rhizosphere harboured more core OTUs (357 OTUs) than the bulk soil (190 OTUs) in the bacterial, archaeal, and fungal communities, although the species richness values in the bulk soil and rhizosphere were similar (Fig. 4). Furthermore, while the number of core OTUs in the rhizosphere and endosphere was small, these OTUs accounted for a large fraction of the OTU abundances in bacterial and archaeal communities (Fig. 4). The bacterial core OTUs that were shared by the rhizosphere and endosphere contained the aforementioned indicator taxa that might exert beneficial effects on plant growth. These results suggest that plants recruit beneficial microorganisms that are abundant in the rhizosphere^5^, which is also supported by the higher homogeneity of microbial communities in the rhizosphere than of those in the other compartments (Fig. 2). In the fungal communities, the number of core OTUs was low (12 OTUs) compared to the number of bacterial core OTUs (374 OTUs), even though the total number of fungal OTUs (4,940 OTUs) was greater than that of bacterial OTUs (3,511 OTUs). This finding indicates that the composition of fungal OTUs was very different between each sample, which is consistent with a previous report in which the similarity of fungal communities between tropical soils was seen to be lower than that of bacterial communities^54^. It is likely that fungal communities are strongly influenced by environmental factors and stochastic assembly processes compared to bacterial communities^55^.

By combining the bacterial, fungal, and archaeal community datasets, co-occurrence network analysis was performed to gain insights into potential relationships between the taxa in the different rhizocompartments. The rhizospheric network exhibited a higher complexity of microbial communities than the bulk soil network, as shown by the greater number of links and higher avgK value (Table 3). This finding is consistent with previous results obtained with the rhizosphere of oat and *Jacobaea vulgaris*, which were analysed by different algorithms, namely, the molecular ecological networks (MENs) based on RMT and SparCC, respectively^30,56^. The complexity of the network found in the rhizosphere is most likely due to continuous changes in the local environment of roots and surrounding soil, such as changes in nutrient availability, pH, moisture, oxygen content, and carbon dioxide levels^16,30^. In contrast to the rhizosphere, microbial communities in the endosphere presented very low species richness, diversity, and network complexity, indicating that the limited number of species were physically separated by plant tissue and/or that the number of microbial interactions with the host plant was much greater than the number of microbe-microbe interactions. Taken together, the results show that the rhizosphere is the most active site of microbe-microbe and plant-microbe interactions. We identified several OTUs as module hubs and connectors that play potential roles in the network structure and suggested that these OTUs are keystone taxa. All potential keystone OTUs detected in the bulk soil, rhizosphere, and endosphere were species of bacteria, even though the microbial networks included bacterial, archaeal, and fungal taxa, which suggests that bacteria might play a key role in linking members of archaea and fungi in the heterogenous microbial communities. As previously reported, we observed that most of the keystone OTUs had relatively low abundances (<0.1%), and these taxa might be important players in the structure and functioning of microbial communities in each rhizocompartment^30,57,58^. To reveal their specific roles, experimental evidences complementing the computational inference carried out is needed. The manipulation of unculturable microorganisms in an experiment is the biggest obstacle we must overcome in the future.

Prediction of gene function profiles using PICRUSt with bacterial 16S rRNA gene sequence data showed that genes associated with membrane transport were the most abundant across the functional categories and were significantly more relatively abundant in the endosphere and rhizosphere, than in bulk soil (Fig. 5). In previous studies, metagenomic analyses of soybean and *J*. *vulgaris* rhizospheres have shown that functional genes associated with membrane transport were more abundant in the rhizosphere than in bulk soil^56,59^. Considering the fact that the rhizosphere and endosphere are regions where microorganisms and plants interact via the uptake and export of various substrates, genes involved in membrane transport potentially play critical roles in interactions with the host plants in these regions. Rhizospheric and endospheric microorganisms take up carbon exuded from plant roots and secrete diverse compounds that have beneficial effects on plant growth and health. Examples of well-known molecules that promote plant growth include indole-3-acetic acid (IAA), which is a phytohormone that regulates plant development; 1-aminocyclopropane-1-carboxylic acid (ACC) deaminase, which reduces the ethylene content by degrading an ethylene precursor; and 2,3-butanediol, which is a volatile organic compound that induces the plant defence system^60,61^. Secondary metabolites with antibiotic activity, including siderophores and lytic enzymes such as chitinase, have been shown to inhibit plant pathogens and cause disease suppression in plants^62,63^. Further metatranscriptomic and metaproteomic research is needed to identify the function of microbiomes associated with plant growth and health.

### Conclusions, limitations, and future directions

We investigated the differences in the microbial communities found in the bulk soil, rhizosphere, and endosphere of tomato plants under natural systems, analysing the diversity, composition, structure, network, and metagenome prediction of the microbial communities. The findings of the present study have several implications. First, the microbial communities in the endosphere, with low species richness and diversity, were highly variable and less influenced by edaphic factors than those in the other rhizocompartments. Second, the taxa that are significantly more relatively abundant in the rhizosphere and endosphere have potentially beneficial effects on plant growth and health. Third, the microbial communities in the rhizosphere exhibited a complex network, while those in the endosphere had a smaller and simpler network consisting of fewer keystone taxa. Lastly, the microbial communities inhabiting the endosphere and rhizosphere possessed more genes involved in membrane transport than those found in bulk soil samples, indicating active exchange of diverse compounds between microorganisms and plants.

Conducting a microbiome study, particularly under natural systems, is challenging because microbiomes are sensitive to surrounding environmental conditions. Although the primary objective of this study was to screen three kingdoms of organisms found in microbial communities of tomato plants under natural conditions and to reveal clear niche differentiation of microbial communities, other factors, such as host genotype, phenotype (e.g., enhanced growth, disease susceptibility/tolerance), developmental stage of plants (sample collection timing), and climatic condition, might have contributed to this differentiation. Thus, future work should put more effort into defining and minimizing variables across samples in order to provide a complementary and more comprehensive picture of microbial communities and contribute to the understanding of the factors that drive the structure of these microbial communities. Furthermore, predicting functional profiles of microbial communities based on 16S rRNA amplicon sequencing data does not provide adequate genomic and functional details of the microbiome, although it is widely used for screening purposes. On the other hand, a metagenomic approach would help determine the functional mechanisms mediating plant–microbe interactions and define the core microbiome of plants. Such information is needed to understand and manage microbial functions in each compartment of the tomato roots.

Looking forward, identifying the distinct features of microbial communities in each compartment of tomato roots under real-world environments will improve the understanding of plant-microbiome interactions, thus contributing to the progress of microbiome engineering and to increased plant productivity in the future.

## Materials and Methods

### Study sites and sample preparation

Tomato plants were sampled from 23 different greenhouses in 7 provinces across South Korea, which has a temperate climate (Supplementary Fig. S1). Information regarding the study sites and tomato cultivars is listed in Supplementary Table S1. The tomatoes were planted under the ground between November and December 2014, and tomato plants in the ripening stage were collected between May and June 2015 in the middle of the harvest season. From each greenhouse, three individual tomato plants (spaced 15 m apart) were randomly collected along with soil from a 30-cm radius and pooled. The tomato samples were brought to a laboratory and separated into bulk soil, rhizospheric soil, and endosphere^8,9^. Loose soil without tomato roots was carefully collected as the bulk soil. Subsequently, the tomato roots were vigorously shaken by hand to remove adherent soil particles. The roots with firmly attached soil were placed into 50-ml tubes containing 0.85% NaCl and shaken vigorously using a shaker (CUTE MIXER CM-1000, EYELA, Japan) for 30 min. After the roots were removed from the tubes, the tubes containing soil and saline water were centrifuged at 8,000 rpm for 15 min. The supernatants were removed, and the remaining soil samples were used as the rhizosphere samples. The roots removed during rhizosphere sample preparation were used as the endosphere samples after sterilization of the root surfaces following a series of washing steps: 70% (v/v) ethanol for 1 min, 3% (v/v) sodium hypochlorite solution for 3 min, 2.5% (w/v) sodium thiosulfate for 5 min, and rinsing the samples five times with sterile water. The sterile root samples were homogenized with liquid nitrogen (LN_2_) and stored with the bulk soil and rhizosphere samples at −80 °C until DNA extraction.

### Soil physicochemical analyses

Prior to analysis of the soil physiochemical properties, the collected bulk soil samples were air dried at room temperature in the shade for three days and passed through a 2-mm sieve. Soil pH and EC values were measured using a pH meter (CyberScan pH 1500, EUTECH, USA) and an EC meter (D-54, Horiba, Japan), respectively, after shaking the soil:water (1:5 w/v) mixture for 30 min at 200 rpm. The OM content was measured using the Wakely and Black method^64^. The TN content was measured by the Kjeldahl method^65^. The available phosphate content was measured by the Bray No. 1 method^66^. The exchangeable Ca^2+^, Mg^2+^, Na^+^ and K^+^ content was determined by extracting the soil samples by the ammonium-acetate (1 N, pH 7.0) infusion method and analyzing the extracts by inductively coupled plasma (ICP) analysis (GBC Integra XL, Australia)^67^.

### DNA extraction and amplicon sequencing

Bulk soils, rhizosphere soils, and homogenized plant materials (0.3 g) were used for DNA extraction using the Power Soil DNA Isolation Kit (MoBio, USA) according to the manufacturer’s instructions in triplicate and subsequently pooled. Prior to DNA extraction, each of three types of soil samples derived from individual tomato plant was separately prepared and pooled together to minimize variation. The extracted DNA was quantified using the Qubit dsDNA BR Assay Kits (Invitrogen, USA). To generate bacterial, archaeal and fungal libraries, universal 16S rRNA gene primers (799F: 5′-AACMGGATTAGATACCCKG-3′ and 1193R: 5′-ACGTCATCCCCACCTTCC-3′), archaeal 16S rRNA gene primers (archaea519F: 5′-CAGCCGCCGCGGTAA-3′ and archaea958R: 5′- YCCGGCGTTGAMTCCAATT-3′), and ITS region primers (ITS3F: 5′-GCATCGATGAAGAACGCAGC-3′ and ITS4R: 5′-TCCTCCGCTTATTGATATGC-3′) were used for bacteria^15,68^, archaea^69^, and fungi^70^, respectively. These target-specific primers were attached to Nextera consensus and adaptor sequences with the forward (5′-TCGRCGGCAGCGTC-AGATGTGTATAAGAGACAG-target sequence-3′) and reverse (5′-GTCTCGTGGGCTCGG-AGATGTGTATAAGAGACAG-target sequence-3′) primers for the first round of PCR amplification with the following conditions: initial denaturation at 94 °C for 3 min, denaturation at 94 °C for 30 s, annealing at 55 °C for 30 s, extension at 72 °C for 30 s and final extension at 72 °C for 5 min. Following initial amplification of the bacterial, archaeal and fungal target sequences, the library size was verified by agarose gel electrophoresis, and the PCR products were cleaned using Agencourt AMPure XP (Beckman Coulter, Inc., USA). A second round of PCR amplification was conducted with primers containing Illumina dual indices and sequencing adapters, namely, S502F (5′-AATGATACGGCGACCACCGAGATCTACAC-55555555-TCGTCGGCAGCGTC-3′) and N701R (5′-CAAGCAGAAGACGGCATACGAGAT-77777777-AGTCTCGTGGGCTCGG-3′), under the same conditions as those used for the first round of PCR. The PCR products were cleaned using Agencourt AMPure XP (Beckman Coulter, Inc., USA) and quantified with the Quant-iT PicoGreen dsDNA Assay Kit (Invitrogen, USA). Purified amplicon libraries were pooled at equimolar concentrations and sequenced with an Illumina MiSeq system by using MiSeq Reagent Kit v3 (Illumina Inc., USA). The bacterial DNA samples were sequenced at the National Instrumentation Center for Environmental Management (NICEM; South Korea), and the archaeal and fungal DNA samples were sequenced at ChunLab Inc. (South Korea). Sequence data are available in the GenBank SRA database under BioProject accession number PRJNA383959.

### Sequence data processing

The sequences obtained from the MiSeq platform were processed using the UPARSE pipeline (ver.9.1.13_i86linux64, www.drive5.com/usearch)^71^. The paired-end reads were merged when the number and ratio of mismatches in the overlap region were less than 10 and 10%, respectively. Low-quality reads that were above the expected error threshold (>1) and short reads (<300 bp) were removed. To minimize the impact of sequencing artefacts, singletons were removed from the datasets^72^. Chimeric sequences were removed using the UCHIME *de novo* algorithm. The remaining high-quality sequences were clustered into OTUs with 97% identity by the UPARSE algorithm. Representative sequences of bacterial and archaeal OTUs were classified using the naïve Bayesian classifier^73^ based on the Ribosomal Database Project (RDP) database^74^ with a 60% confidence threshold. The OTUs affiliated with chloroplasts and archaea were subsequently removed from the bacterial OTU table, and those affiliated with bacteria were removed from the archaeal OTU table. The fungal OTUs were classified with the UNITE database^75^ with a 60% confidence threshold, and OTUs that were assigned to non-fungi including plant and protozoa were removed from the fungal OTU table. Rarefaction curves were generated by Mothur (version 1.29.1; www.mothur.org)^76^. For assessing alpha-diversity indices, sequence reads of bulk soil and rhizosphere samples were rarefied to 20,000 and those of endosphere samples to 4,000, and six indices including coverage, number of OTUs, Chao1, ACE, Shannon, and Inverse Simpson were subsequently calculated using Mothur. Faith’s PD was calculated using the picante package of R 3.3.1 (R Development Core Team, 2014) with a UPGMA phylogenetic tree that was constructed after MUSCLE alignment^77^ of the OTU sequences.

### Statistical analyses

Statistical analyses in this study were performed using R 3.3.1 (R Development Core Team, 2014). The OTU abundances in the dataset were normalized with Hellinger transformation^78^ using the ‘*decostand*’ function of the *vegan* package^79^ in R. Subsequently, NMDS analysis was performed based on the Bray-Curtis dissimilarity matrix using the “*metaMDS*” function of the *vegan* package. PCoA of weighted UniFrac distances of microbial communities was performed using the “beta_diversity.py” script in QIIME with a UPGMA phylogenetic tree that was constructed after MUSCLE alignment^77^. Differences in community structure between rhizocompartments were tested by ANOSIM^80^ using the ‘*anosim*’ function and PERMANOVA^81^ using the ‘*adonis*’ function based on the Bray-Curtis dissimilarity matrix calculated using the “*vegdist*” function within the *vegan* package. The dispersion of microbial communities within each compartment was analysed using the ‘*betadisper*’ function in the *vegan* package. The Mantel test was performed to analyse the correlation between microbial community compositions and soil physicochemical properties. All soil chemical variables except for pH were log-transformed for a normal distribution. The correlations between the Euclidean dissimilarity of the soil physicochemical properties and the Bray-Curtis distance of the microbial community composition were analysed with the ‘*mantel*’ function in the *vegan* package. CCA analysis was conducted using the ‘*cca*’ function of the *vegan* package, and forward selection of environmental variables based on RDA was examined using the ‘*forward*.*sel*’ function of the *packfor* package. To identify the OTUs that were specifically abundant in each compartment, indicator species analysis was conducted using the ‘*multipatt*’ function with the ‘*r*.*g*’ option in the *indispecies* package^82^ and the OTUs with high indicator values (>0.5) and high abundances (>1% relative abundance) were screened from the results. Linear discriminant analysis (LDA) effect size (LEfSe) was performed online (http://huttenhower.sph.harvard.edu/galaxy)^83^. A logarithmic LDA score was set to 3.5 with statistical significance (p < 0.05). Core OTUs that were present in all samples of each rhizocompartment were detected using “get.sharedseqs” function of Mothur program.

### Network analysis

Network analysis was conducted by following the Molecular Ecological Network Analyses (MENA) Pipeline based on RMT at the University of Oklahoma’s Institute for Environmental Genomics web server (http://ieg2.ou.edu/MENA/)^37^. The details of the process are provided in Shi *et al*. (2017). The input datasets contained the relative abundances of OTUs from the 23 samples of each rhizocompartment (bulk soil, rhizosphere, and endosphere). For network construction, the following options were used: OTUs detected in more than half of the samples were used; 0.01 was filled in the blanks with paired valid values; logarithm values were obtained; Spearman’s Rho was used for correlation analysis; and calculation were made by decreasing the cutoff from the top with a Poisson regression only. A similarity threshold was selected automatically by the RMT-based approach to define the adjacency matrix. The modularity of the network was calculated using the greedy modularity optimization method. Subsequently, the within-module connectivity (Zi) and among-module connectivity (Pi) of each node were examined, and topological roles were classified based on the values of Zi (2.5) and Pi (0.62). Networks were visualized using Gephi^84^.

### Prediction of bacterial functional gene profiles

Metagenomic functional gene profiles were predicted using Phylogenetic Investigation of Communities by Reconstruction of Unobserved States (PICRUSt) with bacterial 16S rRNA gene sequences^85^. Bacterial 16S rRNA gene sequences were picked using the pick_closed_reference_otus.py script in QIIME based on the Greengenes database (ver. 13.8). Chloroplast sequences were removed using the filter_taxa_from_otu_table.py script in QIIME. The filtered biom file was uploaded and analysed with the online Galaxy version of PICRUSt (http://galaxy.morganlangille.com/) based on the KEGG pathway database.

## Supplementary information

Tomato microbiome_Supplementary information

Supplementary data S1. Core OTUs

## References

[1] Yang J, Kloepper JW, Ryu CM (2009). Rhizosphere bacteria help plants tolerate abiotic stress. Trends Plant Sci.

[2] Berendsen RL, Pieterse CMJ, Bakker P (2012). The rhizosphere microbiome and plant health. Trends Plant Sci..

[3] Bais HP, Park S-W, Weir TL, Callaway RM, Vivanco JM (2004). How plants communicate using the underground information superhighway. Trends Plant Sci..

[4] Philippot L, Raaijmakers JM, Lemanceau P, van der Putten WH (2013). Going back to the roots: the microbial ecology of the rhizosphere. Nat. Rev. Microbiol..

[5] Bulgarelli D, Schlaeppi K, Spaepen S, van Themaat EVL, Schulze-Lefert P (2013). Structure and functions of the bacterial microbiota of plants. Annu. Rev. Plant Biol..

[6] Compant S, Clement C, Sessitsch A (2010). Plant growth-promoting bacteria in the rhizo- and endosphere of plants: Their role, colonization, mechanisms involved and prospects for utilization. Soil Biol. Biochem..

[7] Gottel NR (2011). Distinct microbial communities within the endosphere and rhizosphere of *Populus deltoides* roots across contrasting soil types. Appl. Environ. Microb..

[8] Lundberg DS (2012). Defining the core *Arabidopsis thaliana* root microbiome. Nature.

[9] Bulgarelli D (2012). Revealing structure and assembly cues for *Arabidopsis* root-inhabiting bacterial microbiota. Nature.

[10] Beckers B, Op De Beeck M, Weyens N, Boerjan W, Vangronsveld J (2017). Structural variability and niche differentiation in the rhizosphere and endosphere bacterial microbiome of field-grown poplar trees. Microbiome.

[11] Edwards J (2015). Structure, variation, and assembly of the root-associated microbiomes of rice. Proc. Natl. Acad. Sci. USA.

[12] Coleman-Derr D (2016). Plant compartment and biogeography affect microbiome composition in cultivated and native *Agave* species. New Phytol..

[13] Fonseca-Garcia C (2016). The Cacti microbiome: interplay between habitat-filtering and host-specificity. Front. Microbiol..

[14] Mora-Ruiz Mdel, R., Font-Verdera, F., Orfila, A., Rita, J. & Rossello-Mora, R. Endophytic microbial diversity of the halophyte *Arthrocnemum macrostachyum* across plant compartments. *FEMS Microbiol*. *Ecol*. **92** (2016). 10.1093/femsec/fiw145 27353659

[15] Schlaeppi K, Dombrowski N, Oter RG, van Themaat EVL, Schulze-Lefert P (2014). Quantitative divergence of the bacterial root microbiota in *Arabidopsis thaliana* relatives. Proc. Natl. Acad. Sci. USA.

[16] Chaparro JM, Badri DV, Vivanco JM (2014). Rhizosphere microbiome assemblage is affected by plant development. ISME J..

[17] Peiffer JA (2013). Diversity and heritability of the maize rhizosphere microbiome under field conditions. Proc. Natl. Acad. Sci. USA.

[18] Fierer N, Jackson RB (2006). The diversity and biogeography of soil bacterial communities. Proc. Natl. Acad. Sci. USA.

[19] Griffiths RI (2011). The bacterial biogeography of British soils. Environ. Microbiol..

[20] Docherty KM (2015). Key edaphic properties largely explain temporal and geographic variation in soil microbial communities across four biomes. PLoS One.

[21] Zhang T, Wang NF, Liu HY, Zhang YQ, Yu LY (2016). Soil pH is a key determinant of soil fungal community composition in the Ny-Alesund Region, Svalbard (High. Arctic). Front. Microbiol..

[22] Jiang L (2016). Exploring the influence of environmental factors on bacterial communities within the rhizosphere of the Cu-tolerant plant, *Elsholtzia splendens*. Sci. Rep..

[23] Buee M, De Boer W, Martin F, van Overbeek L, Jurkevitch E (2009). The rhizosphere zoo: an overview of plant-associated communities of microorganisms, including phages, bacteria, archaea, and fungi, and of some of their structuring factors. Plant Soil.

[24] Mendes R, Garbeva P, Raaijmakers JM (2013). The rhizosphere microbiome: significance of plant beneficial, plant pathogenic, and human pathogenic microorganisms. FEMS Microbiol. Rev..

[25] Vinale F (2008). Trichoderma–plant–pathogen interactions. Soil Biol. Biochem..

[26] Fravel D, Olivain C, Alabouvette C (2003). *Fusarium oxysporum* and its biocontrol. New Phytologist.

[27] Bonfante P, Anca IA (2009). Plants, mycorrhizal fungi, and bacteria: a network of interactions. Annu. Rev. Microbiol..

[28] Chen XP, Zhu YG, Xia Y, Shen JP, He JZ (2008). Ammonia-oxidizing archaea: important players in paddy rhizosphere soil?. Environ. Microbiol..

[29] Gamalero E, Berta G, Massa N, Glick BR, Lingua G (2008). Synergistic interactions between the ACC deaminase-producing bacterium *Pseudomonas putida* UW4 and the AM fungus *Gigaspora rosea* positively affect cucumber plant growth. FEMS Microbiol. Ecol..

[30] Shi S (2016). The interconnected rhizosphere: high network complexity dominates rhizosphere assemblages. Ecol. Lett..

[31] Romero FM, Marina M, Pieckenstain FL (2014). The communities of tomato (*Solanum lycopersicum* L.) leaf endophytic bacteria, analyzed by 16S-ribosomal RNA gene pyrosequencing. FEMS Microbiol. Lett..

[32] Ottesen, A. R. *et al*. Baseline survey of the anatomical microbial ecology of an important food plant: *Solanum lycopersicum* (tomato). *BMC Microbiol*. **13** (2013). 10.1186/1471-2180-13-114 PMC3680157 23705801

[33] Allard SM (2016). *Solanum lycopersicum* (tomato) hosts robust phyllosphere and rhizosphere bacterial communities when grown in soil amended with various organic and synthetic fertilizers. Sci. Total Environ..

[34] Larousse M (2017). Tomato root microbiota and *Phytophthora parasitica*-associated disease. Microbiome.

[35] Tian BY, Cao Y, Zhang KQ (2015). Metagenomic insights into communities, functions of endophytes, and their associates with infection by root-knot nematode, *Meloidogyne incognita*, in tomato roots. Sci. Rep..

[36] Liu J (2014). High throughput sequencing analysis of biogeographical distribution of bacterial communities in the black soils of northeast China. Soil Biol. Biochem..

[37] Deng Y (2012). Molecular ecological network analyses. BMC Bioinformatics.

[38] Hardoim PR, van Overbeek LS, van Elsas JD (2008). Properties of bacterial endophytes and their proposed role in plant growth. Trends Microbiol..

[39] Kim JM (2016). Soil pH and electrical conductivity are key edaphic factors shaping bacterial communities of greenhouse soils in Korea. J. Microbiol..

[40] Goldmann K (2016). Divergent habitat filtering of root and soil fungal communities in temperate beech forests. Sci. Rep..

[41] Feeney KA (2016). Daily magnesium fluxes regulate cellular timekeeping and energy balance. Nature.

[42] Jiang F., Chen L., Belimov A. A., Shaposhnikov A. I., Gong F., Meng X., Hartung W., Jeschke D. W., Davies W. J., Dodd I. C. (2012). Multiple impacts of the plant growth-promoting rhizobacterium Variovorax paradoxus 5C-2 on nutrient and ABA relations of Pisum sativum. Journal of Experimental Botany.

[43] Sousa JAdJ, Olivares FL (2016). Plant growth promotion by streptomycetes: ecophysiology, mechanisms and applications. Chem. Biol. Technol. Agric..

[44] van Rhijn P, Vanderleyden J (1995). The Rhizobium-plant symbiosis. Microbiol. Rev..

[45] Preston GM (2004). Plant perceptions of plant growth-promoting *Pseudomonas*. Philos. Trans R. Soc. Lond. B. Biol. Sci..

[46] Jha C. K. *et al*. In *Bacteria in Agrobiology: Plant Growth Responses* (ed. Maheshwari, D.) 159–182 (Springer, 2011).

[47] Chaerani R, Voorrips RE (2006). Tomato early blight (*Alternaria solani*): the pathogen, genetics, and breeding for resistance. J. Gen. Plant Pathol..

[48] Zivkovic S (2010). Morphological and molecular identification of *Colletotrichum acutatum* from tomato fruit. Pestic. Phytomed. (Belgrade).

[49] da Costa PB (2014). A model to explain plant growth promotion traits: a multivariate analysis of 2,211 bacterial isolates. PLoS One.

[50] Hamayun M (2009). *Chrysosporium pseudomerdarium* produces gibberellins and promotes plant growth. J. Microbiol..

[51] Zhang S, Gan Y, Xu B (2016). Application of plant-growth-promoting fungi *Trichoderma longibrachiatum* T6 enhances tolerance of wheat to salt stress through improvement of antioxidative defense system and gene expression. Front. Plant Sci..

[52] Hung, R. & Lee Rutgers, S. In *New and Future Developments in Microbial Biotechnology and Bioengineering* (ed. Vijai Kumar Gupta) 223–227 (Elsevier, 2016).

[53] Shade A, Handelsman J (2012). Beyond the Venn diagram: the hunt for a core microbiome. Environ. Microbiol..

[54] de Gannes V, Eudoxie G, Bekele I, Hickey WJ (2015). Relations of microbiome characteristics to edaphic properties of tropical soils from Trinidad. Front. Microbiol..

[55] Powell JR (2015). Deterministic processes vary during community assembly for ecologically dissimilar taxa. Nat. Commun..

[56] Yan Y, Kuramae EE, de Hollander M, Klinkhamer PG, van Veen JA (2017). Functional traits dominate the diversity-related selection of bacterial communities in the rhizosphere. ISME J..

[57] Lyons KG, Schwartz MW (2001). Rare species loss alters ecosystem function - invasion resistance. Ecol. Lett..

[58] Pester M, Bittner N, Deevong P, Wagner M, Loy A (2010). A ‘rare biosphere’ microorganism contributes to sulfate reduction in a peatland. ISME J..

[59] Mendes LW, Kuramae EE, Navarrete AA, van Veen JA, Tsai SM (2014). Taxonomical and functional microbial community selection in soybean rhizosphere. ISME J..

[60] Yi HS (2016). Impact of a bacterial volatile 2,3-butanediol on *Bacillus subtilis* rhizosphere robustness. Front. Microbiol..

[61] Spaepen, S. & Vanderleyden, J. Auxin and plant-microbe interactions. *Cold Spring Harb*. *Perspect*. *Biol*. **3** (2011). 10.1101/cshperspect.a001438 PMC3062209 21084388

[62] Premachandra, D., Hudek, L. & Brau, L. Bacterial modes of action for enhancing of plant growth. *J*. *Biotechnol*. *Biomater* **6** (2016).

[63] Zhang Z, Yuen GY, Sarath G, Penheiter AR (2001). Chitinases from the plant disease biocontrol agent, *Stenotrophomonas maltophilia* C3. Phytopathology.

[64] Allison, L. E. In *Methods of Soil Analysis*. *Part 2*. *Chemical and Microbiological* Properties (ed. Norman, A. G.) 1367–1378 (American Society of Agronomy, Soil Science Society of America, 1965).

[65] Bremner J (1960). Determination of nitrogen in soil by the Kjeldahl method. J. Agric. Sci..

[66] Bray RH, Kurtz LT (1945). Determination of total, organic, and available forms of phosphorus in soils. Soil Science Annu. Rev. Microbiol..

[67] Joa JH, Weon HY, Hyun HN, Jeun YC, Koh SW (2014). Effect of long-term different fertilization on bacterial community structures and diversity in citrus orchard soil of volcanic ash. J. Microbiol..

[68] Bulgarelli D (2015). Structure and Function of the Bacterial Root Microbiota in Wild and Domesticated Barley. Cell Host Microbe.

[69] DeLong EF (1992). Archaea in coastal marine environments. Proc. Natl. Acad. Sci. USA.

[70] Bellemain E (2010). ITS as an environmental DNA barcode for fungi: an in silico approach reveals potential PCR biases. BMC Microbiol..

[71] Edgar RC (2013). UPARSE: highly accurate OTU sequences from microbial amplicon reads. Nat. Methods.

[72] Dickie IA (2010). Insidious effects of sequencing errors on perceived diversity in molecular surveys. New Phytol..

[73] Wang Q, Garrity GM, Tiedje JM, Cole JR (2007). Naive Bayesian classifier for rapid assignment of rRNA sequences into the new bacterial taxonomy. Appl. Environ. Microb..

[74] Cole JR (2014). Ribosomal Database Project: data and tools for high throughput rRNA analysis. Nucleic Acids Res..

[75] Koljalg U (2013). Towards a unified paradigm for sequence-based identification of fungi. Mol. Ecol..

[76] Schloss PD (2009). Introducing mothur: open-source, platform-independent, community-supported software for describing and comparing microbial communities. Appl. Environ. Microbiol..

[77] Edgar RC (2004). MUSCLE: multiple sequence alignment with high accuracy and high throughput. Nucleic Acids Res..

[78] Legendre P, Gallagher ED (2001). Ecologically meaningful transformations for ordination of species data. Oecologia.

[79] Oksanen J (2013). Vegan: Community Ecology Package. R package version.

[80] Clarke KR (1993). Nonparametric multivariate analyses of changes in community structure. Aust. J. Ecol..

[81] Anderson MJ (2001). A new method for non-parametric multivariate analysis of variance. Austral. Ecol..

[82] De Caceres M, Legendre P (2009). Associations between species and groups of sites: indices and statistical inference. Ecology.

[83] Segata N (2011). Metagenomic biomarker discovery and explanation. Genome biol..

[84] Bastian M., Heymann S. & Jacomy, M. Gephi: an open source software for exploring and manipulating networks. *International AAAI Conference on Weblogs and Social Media* (2009).

[85] Langille MG (2013). Predictive functional profiling of microbial communities using 16S rRNA marker gene sequences. Nat. Biotechnol..

