# Supplementary material for: A preliminary examination of bacterial, archaeal, and fungal communities inhabiting different rhizocompartments of tomato plants under real-world environments
Source: Sci Rep. 2019 Jun 26;9:9300. doi: 10.1038/s41598-019-45660-8 (PMC6594962; doi:10.1038/s41598-019-45660-8)
Supplement: Supplementary file 1 — Tomato microbiome_Supplementary information [file 41598_2019_45660_MOESM1_ESM.pdf]

Supplementary Information

A preliminary examination of bacterial, archaeal, and fungal communities inhabiting different rhizocompartments of tomato plants under real-world environments

Shin Ae Lee[1], Yiseul Kim[1], Jeong Myeong Kim[1], Bora Chu[1], Jae-Ho Joa[2], Mee Kyung Sang[1], Jaekyeong Song[1], Hang-Yeon Weon[1]*

[1]Agricultural Microbiology Division, National Institute of Agricultural Sciences, Rural Development Administration (RDA), Wanju 55365, South Korea

[2]Research Institute of Climate Change and Agriculture, National Institute of Horticultural & Herbal Science, RDA, Jeju 63240, South Korea

***Correspondign Author:** Hang-Yeon Weon (why@korea.kr)

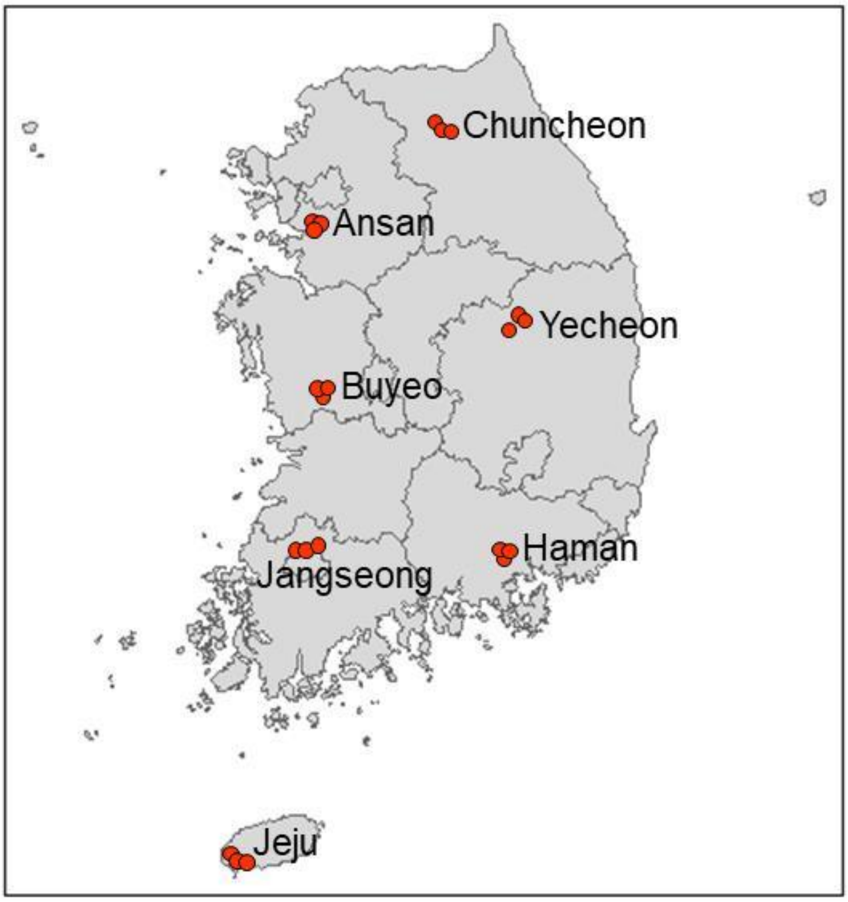

SUPPLEMENTARY FIGURE S1. Map depicting 23 sampling locations for this study.

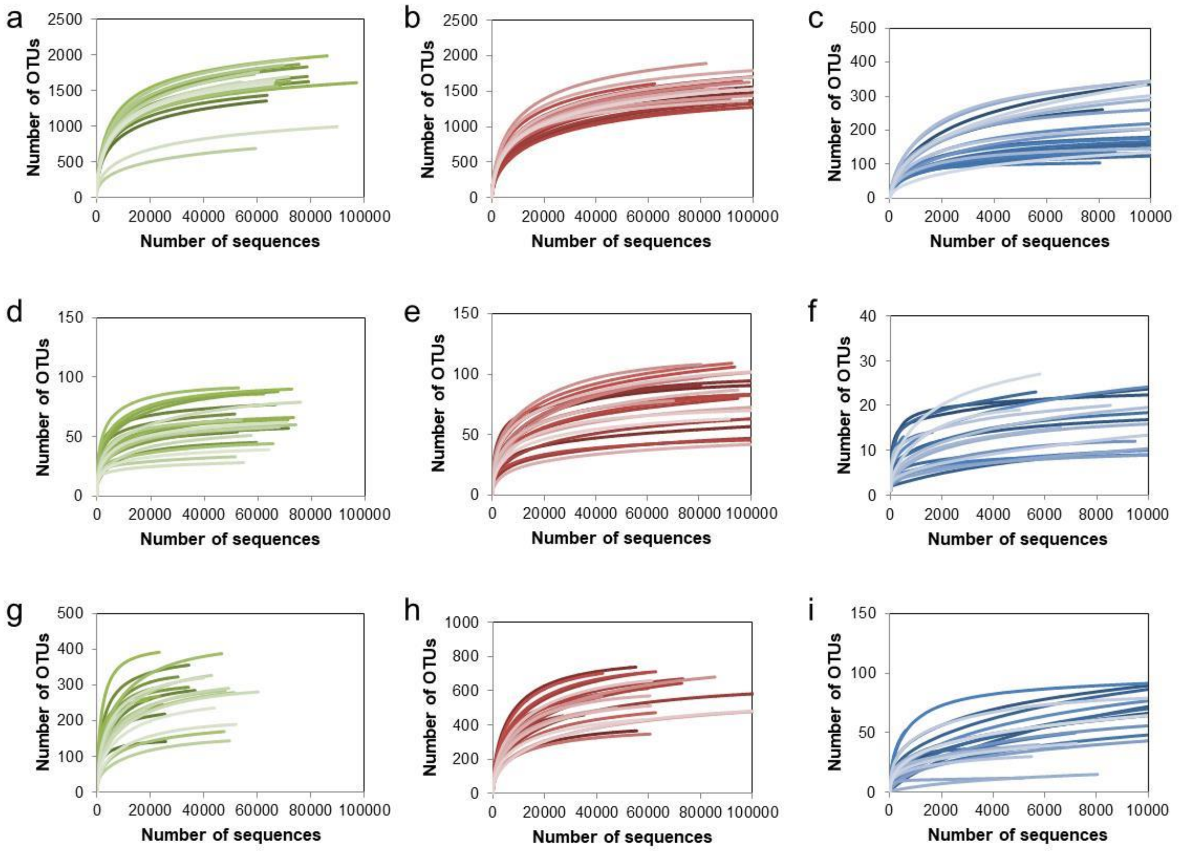

SUPPLEMENTARY FIGURE S2. Rarefaction curves of individual samples according to microorganisms and root compartments. The samples collected from bulk soil (a, d, and g), rhizosphere (b, e, and h), and endosphere (c, f, and i) are amplified with bacterial (a, b, and c), archaeal (d, e, and f), and fungal (g, h, and i) specific primers.

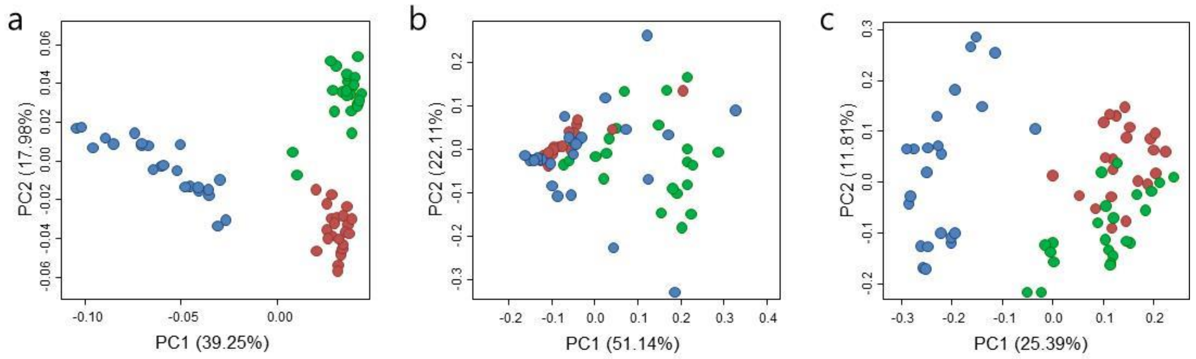

SUPPLEMENTARY FIGURE S3. Principal Coordinates Analysis (PCoA) of weighted UniFrac distances of bacterial (a), archaeal (b), and fungal (c) communities. The colors of the dots denote the compartments of the samples: bulk soil (green), rhizosphere (red), and endosphere (blue).

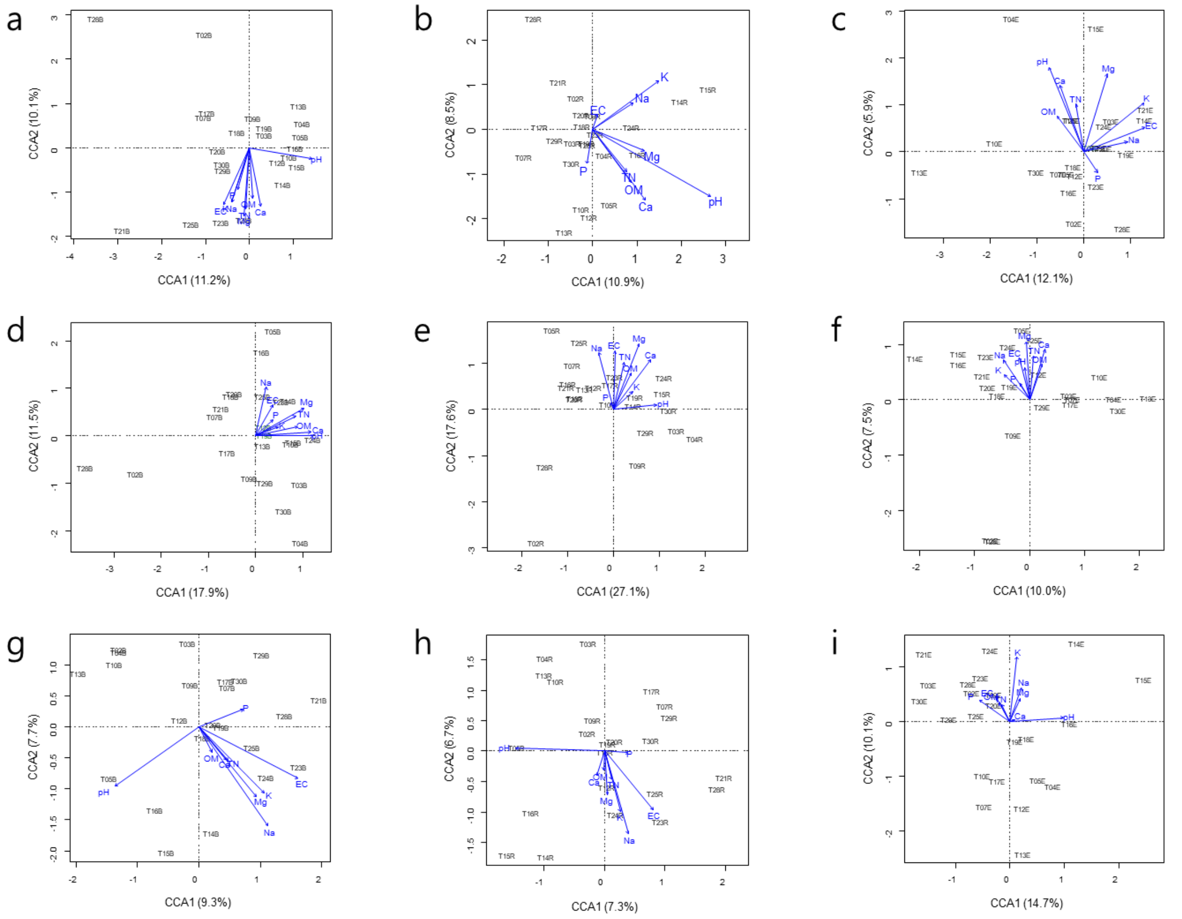

SUPPLEMENTARY FIGURE S4. Canonical correspondence analysis (CCA). Biplots shows the correlations between soil edaphic factors (blue) and the bacterial (a, b, and c), archaeal (d, e, and f), and fungal (g, h, and i) communities in the bulk soil (a, d, and g), rhizosphere (b, e, and h), and endosphere (c, f, and i) samples.

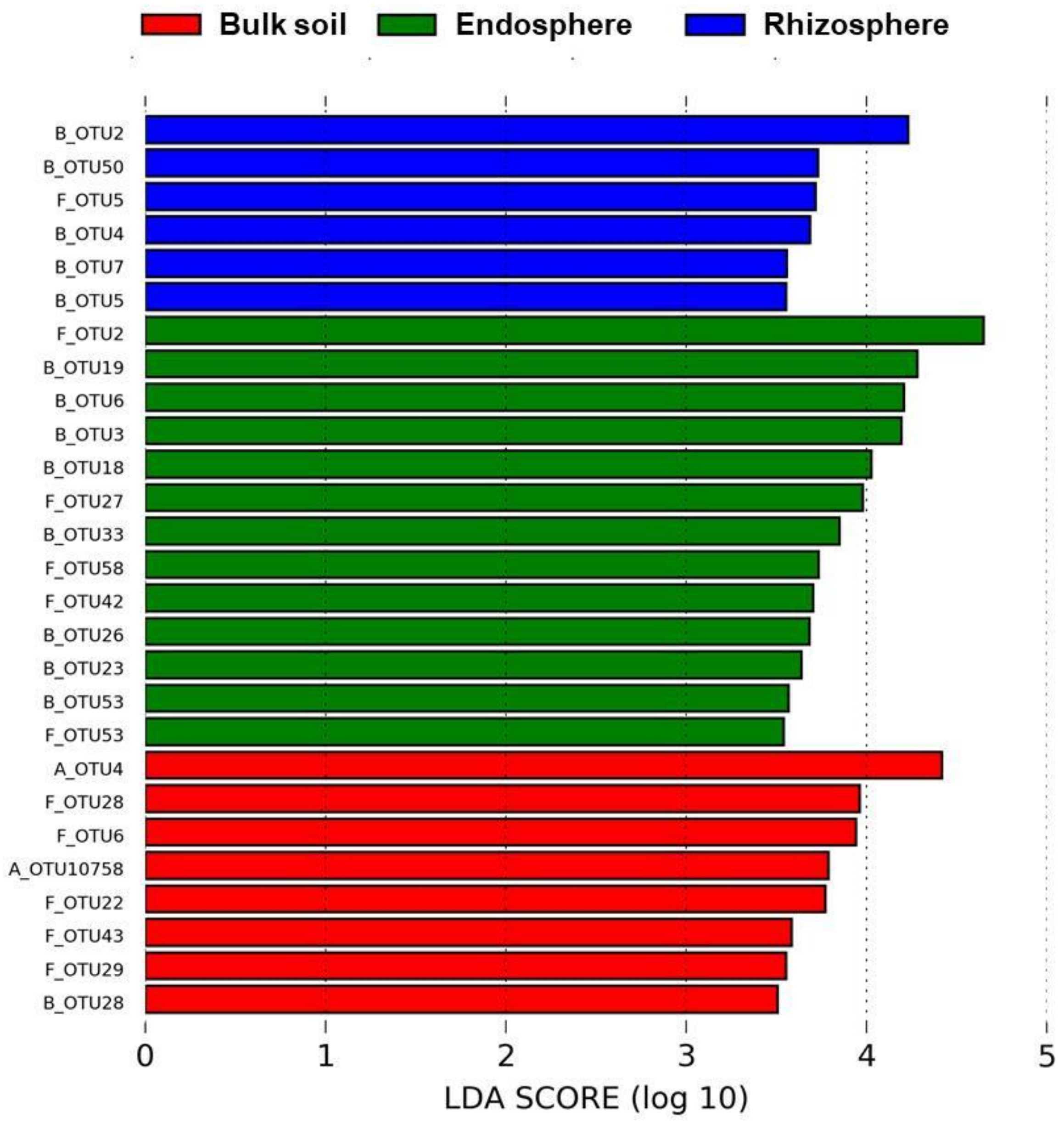

SUPPLEMENTARY FIGURE S5. The Linear discriminant analysis (LDA) Effect Size (LEfSe) analysis showing discriminative OTUs among different rhizocompartments. Relative abundance is significant when $P < 0.05$, logarithmic LDA score ≥3.5.

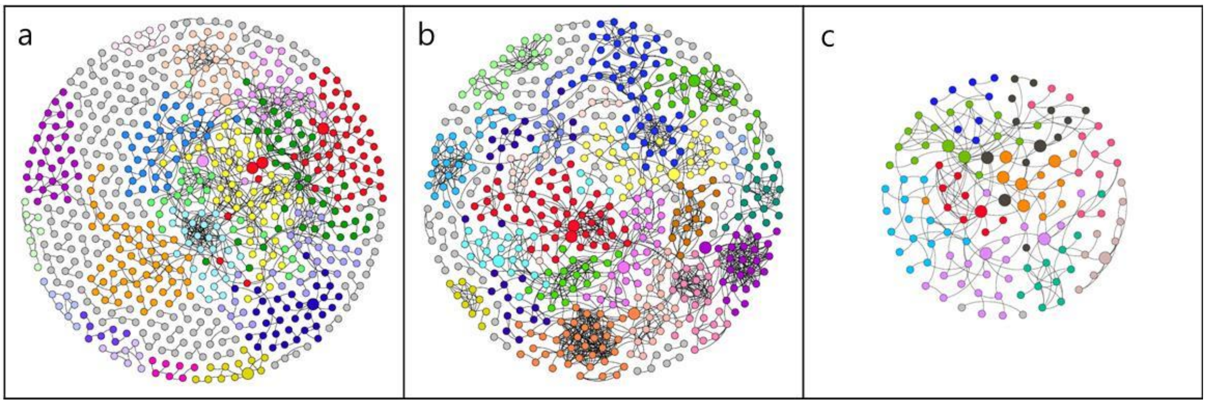

SUPPLEMENTARY FIGURE S6. The submodule structure of microbial network in the bulk soil (a), rhizosphere (b), and endosphere (c) based on the fast greedy modularity optimization method. Nodes represent microbial OTUs and the color denotes different modules.

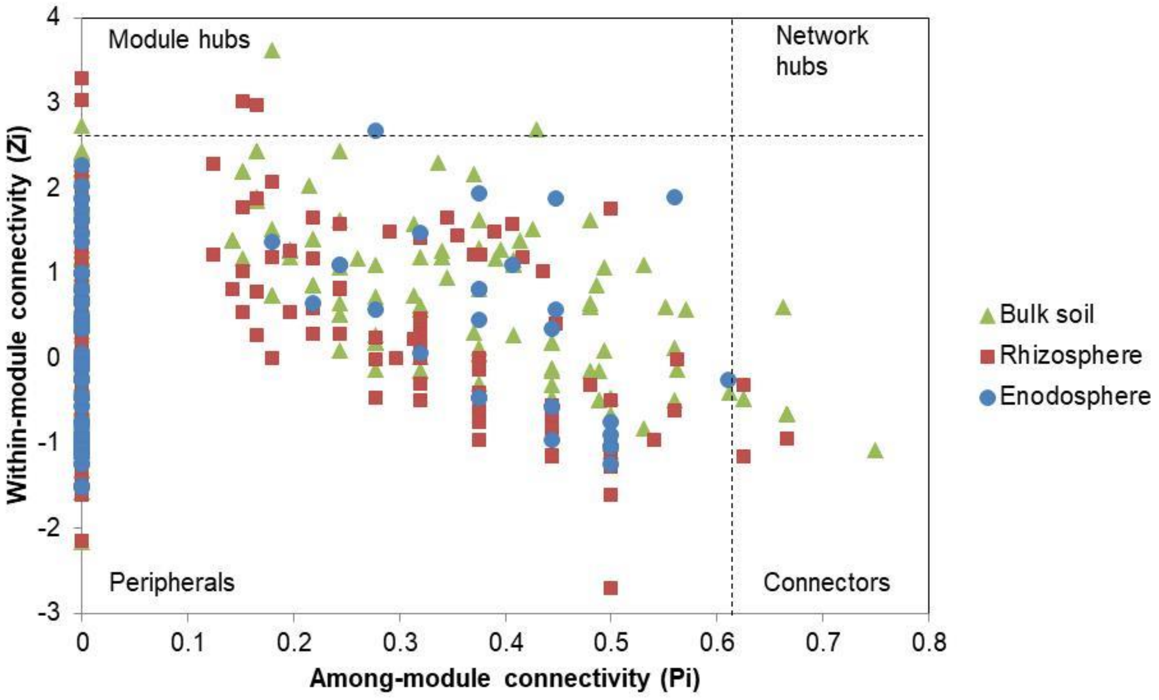

SUPPLEMENTARY FIGURE S7. ZP-plot classifying nodes to module-based topological roles. Each dot represents an OTU in the bulk soil, rhizosphere or endosphere. The threshold values of Zi and Pi for categorizing OTUs as module hubs and connecters were 2.5 and 0.62, respectively.

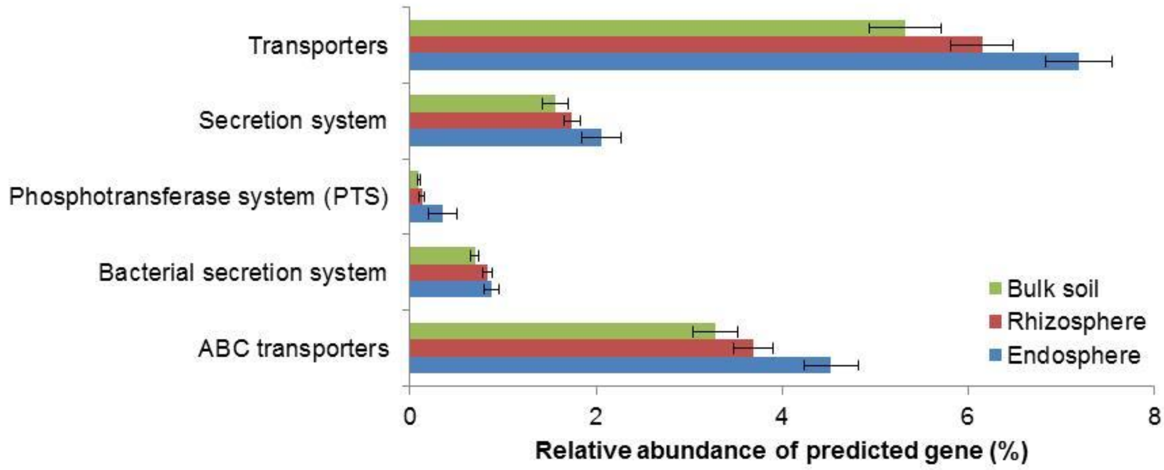

SUPPLEMENTARY FIGURE S8. Functional gene profiles based on KEGG database at level 3 belonging to membrane transport predicted using PICRUSt.

SUPPLEMENTARY TABLE S1. The sample information about designation, collected geographical regions, and cultivars of tomato.

Sample ID	District	Coordinates	Cultivar
T02	Jeju	33°17'45.78"N 126°12'32.06"E	Cutie
T03	Jeju	33°14'04.63"N 126°14'25.36"E	Cutie
T04	Jeju	33°16'06.75"N 126°16'04.86"E	Deachu
T05	Jangseong	35°14'56.05"N 126°50'41.80"E	Altorang
T07	Jangseong	35°14'58.89"N 126°46'39.36"E	Deachu
T09	Jangseong	35°15'59.05"N 126°55'38.83"E	Unicorn
T10	Haman	35°14'57.64"N 128°21'17.49"E	Shabang
T12	Haman	35°14'53.85"N 128°21'16.99"E	Baipass
T13	Haman	35°14'35.67"N 128°21'24.45"E	Baipass
T14	Buyeo	36°17'30.19"N 126°55'58.12"E	Dotaerang-dia
T15	Buyeo	36°17'29.26"N 126°55'58.71"E	Dotaerang-sola
T16	Buyeo	36°17'36.34"N 126°55'54.19"E	Dotaerang-dia
T17	Buyeo	36°18'03.58"N 126°55'49.76"E	Dotaerang-sola
T18	Buyeo	36°17'50.13"N 126°55'28.01"E	Dotaerang-sola
T19	Yecheon	36°42'44.19"N 128°30'07.14"E	Dabok
T20	Yecheon	36°42'03.25"N 128°29'55.96"E	Dabok
T21	Yecheon	36°35'50.08"N 128°23'45.43"E	Alexander
T23	Ansan	37°20'28.48"N 126°51'27.43"E	Rapido
T24	Ansan	37°17'12.81"N 126°52'23.19"E	Deachu
T25	Ansan	37°17'50.20"N 126°53'29.63"E	Mamirio
T28	Chuncheon	37°56'02.63"N 127°46'12.40"E	Heongnog-339
T29	Chuncheon	37°55'53.00"N 127°45'47.81"E	Unicorn
T30	Chuncheon	37°55'35.28"N 127°45'13.16"E	Rapido

SUPPLEMENTARY TABLE S2. Alpha-diversity estimates of the bacterial, archaeal, and fuangal communities in different rhizocompartments. Sequence reads of bulk soil and rhizosphere samples were rarefied to 20,000 and those of endosphere samples to 4,000. Values are average ± standard deviation.

Kingdom	Rhozocompartment	Coverage (%)	No. of OTUs	Chao1	ACE	Shannon	Inverse Simpson
Bacteria	Bulk soil	98.44	1394.52 ± 265.00	1659.74 ± 287.39	1637.50 ± 274.92	6.08 ± 0.49	203.18 ± 81.60
	Rhizosphere	98.43	1362.04 ± 139.45	1625.76 ± 160.31	1609.19 ± 148.92	5.96 ± 0.34	159.58 ± 80.11
	Endosphere	98.76	180.82 ± 62.40	222.76 ± 88.80	230.92 ± 94.03	3.38 ± 0.50	14.86 ± 8.08
Archaea	Bulk soil	99.95	58.09 ± 16.69	66.15 ± 19.08	64.87 ± 16.60	2.14 ± 0.36	5.77 ± 2.12
	Rhizosphere	99.92	74.57 ± 18.15	87.85 ± 24.83	88.15 ± 22.02	1.86 ± 0.37	4.26 ± 1.68
	Endosphere	99.92	14.74 ± 6.38	16.95 ± 8.14	21.26 ± 11.79	1.19 ± 0.45	2.85 ± 1.22
Fungi	Bulk soil	99.78	257.64 ± 65.38	282.21 ± 68.15	287.09 ± 67.54	3.02 ± 0.47	9.04 ± 4.64
	Rhizosphere	99.45	504.83 ± 105.45	581.38 ± 120.16	591.40 ± 123.59	3.93 ± 0.52	20.38 ± 10.99
	Endosphere	99.63	46.38 ± 18.77	64.85 ± 28.48	75.51 ± 37.98	1.51 ± 0.83	3.86 ± 3.38

SUPPLEMENTARY TABLE S3. Chemical properties of bulk soils used in this study. Abbreviations: EC, electrical conductivity; OM, organic matter; TN, total nitrogen; Avail., available.

Sample ID	pH (1:5)	EC (dS/m)	OM (g/kg)	TN (%)	Avail. P_2O_5 (mg/kg)	Exchangeable cation ($cmol_c$/kg)			
						K^+	Ca^{2+}	Mg^{2+}	Na^+
T02	6.5	0.10	24.9	0.12	634.8	0.99	3.1	0.7	0.1
T03	7.2	0.51	38.5	0.19	670.5	1.42	6.1	2.8	0.1
T04	7.6	0.26	26.4	0.15	190.5	0.83	8.9	2.4	0.1
T05	7.1	1.05	44.4	0.26	399.4	0.89	7.0	3.4	0.7
T07	5.8	1.50	23.7	0.16	471.7	0.49	6.9	2.8	0.6
T09	7.0	0.41	26.1	0.14	780.4	1.23	6.0	2.0	0.2
T10	7.2	0.48	53.5	0.31	836.3	1.27	10.8	3.0	0.3
T12	6.6	2.35	47.0	0.33	709.9	0.75	17.2	2.7	0.8
T13	6.9	0.32	44.4	0.21	556.9	0.48	9.8	2.2	0.3
T14	7.5	1.42	40.6	0.25	750.2	3.94	9.7	4.3	1.1
T15	7.8	1.06	39.1	0.23	98.9	4.34	10.4	4.7	1.1
T16	7.2	0.81	35.6	0.20	858.3	1.39	7.5	3.1	0.9
T17	6.1	1.05	17.8	0.13	302.3	0.62	6.0	2.8	0.4
T18	6.6	0.80	22.5	0.15	737.4	0.99	5.9	2.8	0.6
T19	6.6	1.28	21.9	0.14	558.8	0.67	7.3	2.6	0.4
T20	6.6	0.95	29.7	0.20	749.3	1.65	5.7	3.3	0.6
T21	5.9	4.04	54.0	0.49	949.0	7.33	10.7	6.8	1.8
T23	6.4	3.39	53.5	0.36	1319.0	3.79	16.0	6.2	1.9
T24	6.9	2.95	64.8	0.42	917.8	6.55	17.2	7.1	1.5
T25	6.3	4.00	66.9	0.55	754.8	5.46	16.3	7.7	2.3
T28	4.8	0.94	12.1	0.07	106.3	1.13	2.8	1.3	0.6
T29	6.4	1.38	35.6	0.23	1031.4	1.36	10.9	3.1	0.4
T30	6.3	1.86	72.6	0.40	807.0	3.58	17.6	4.3	0.7

SUPPLEMENTARY TABLE S4. Environmental variables related to bacterial, archaeal, and fungal communities. Variables were selected by using forward selection based on RDA. Cumulative adjusted R^2 values of each category were depicted in bold. Abbreviations: EC, electrical conductivity; OM, organic matter.

Domain	Compartment	Variable	R^2_{adj}	Cum R^2_{adj}	*F*	*P*
Bacteria	Bulk soil	pH	0.115	0.115	3.867	0.001
		EC	0.102	0.217	3.750	0.001
		K^+	0.035	0.253	1.960	0.005
		Ca^{2+}	0.026	**0.279**	1.686	0.025
	Rhizosphere	K^+	0.050	0.050	2.176	0.002
		OM	0.060	0.111	2.422	0.001
		pH	0.055	0.166	2.338	0.003
		Na^+	0.044	**0.211**	2.071	0.005
	Endosphere	OM	0.042	0.042	1.953	0.007
		K^+	0.029	**0.071**	1.670	0.029
Archaea	Bulk soil	pH	0.110	0.110	3.743	0.001
		Mg^{2+}	0.073	**0.184**	2.904	0.001
	Rhizosphere	pH	0.120	**0.120**	4.009	0.005
	Endosphere	No variables selected				
Fungi	Bulk soil	EC	0.952	0.095	3.315	0.001
		Na^+	0.035	0.130	1.849	0.010
		Ca^{2+}	0.032	**0.163**	1.788	0.014
	Rhizosphere	Na^+	0.045	0.045	2.049	0.006
		pH	0.032	**0.078**	1.743	0.006
	Endosphere	K^+	0.076	**0.076**	2.826	0.014

SUPPLEMENTARY TABLE S5. The list of OTUs identified as module hubs and connectors. Abbreviations: B_OTU, bacterial OTU; F_OTU, fungal OTU; A_OTU, archaeal OTUs; B, bulk soil; R, rhizosphere; E, endosphere.

Compartment	Topological role	OTU	Zi	Pi	Phylum/Class	Family/Genus	Relative abundance (%)		
							B	R	E
Bulk soil	Module hub	B_OTU97	2.73	0	*Gemmatimonadetes*	*Gemmatimonas*	0.032	0.022	0
		B_OTU3126	2.69	0.43	*Firmicutes*	*Tumebacillus*	0.004	0.005	0
		B_OTU2155	3.62	0.18	*Proteobacteria*	Unclassified	0.003	0.001	0
	Connector	B_OTU1544	-0.653	0.666	*Planctomycetes*	*Planctomyces*	0.002	0.008	0
		B_OTU1390	-1.084	0.75	*Actinobacteria*	*Leucobacter*	0.009	0.009	0.001
		B_OTU1444	-0.484	0.625	*Alphaproteobacteria*	*Rhodospirillaceae*	0.003	0.009	0
		B_OTU1324	-0.653	0.666	*Candidatus_Saccharibacteria*	Unclassified	0.003	0.007	0.009
		B_OTU1377	-0.484	0.625	*Proteobacteria*	Unclassified	0.004	0.004	0
		B_OTU2474	0.609	0.663	*Elusimicrobia*	*Elusimicrobium*	0.008	0.001	0.001
		B_OTU1707	-0.653	0.666	*Proteobacteria*	Unclassified	0.003	0.004	0.001
		B_OTU2008	-0.653	0.666	*Firmicutes*	*Bacillaceae*	0.003	0.002	0
		B_OTU1968	-0.653	0.666	*Proteobacteria*	*Xanthomonadaceae*	0.003	0.001	0
		B_OTU2347	-0.653	0.666	*Firmicutes*	Unclassified	0.003	0.001	0
		B_OTU2201	-0.653	0.666	Unclassified	Unclassified	0.004	0	0
		B_OTU3456	-0.653	0.666	*Actinobacteria*	*Micromonosporaceae*	0.002	0.001	0
Rhizosphere	Module hub	B_OTU546	3.034	0	*Actinobacteria*	Unclassified	0.028	0.020	0.001
		B_OTU831	2.978	0.165	*Bacteroidetes*	*Rhodothermaceae*	0.041	0.005	0
		B_OTU1397	3.03	0.152	*Proteobacteria*	*Rhodospirillaceae*	0.011	0.004	0.002
		B_OTU1045	3.291	0	*Proteobacteria*	Unclassified	0.014	0.005	0
		B_OTU2002	3.034	0	Unclassified	Unclassified	0.015	0.004	0

		B_OTU809	-0.943	0.666	Unclassified	Unclassified	0.054	0.004	0
	Connector	B_OTU3314	-0.309	0.625	*Proteobacteria*	Unclassified	0.016	0.004	0.002
		B_OTU1089	-1.158	0.625	*Ignavibacteriae*	*Ignavibacterium*	0.007	0.005	0.001
Endosphere	Module hub	B_OTU870	2.673	0.277	*Firmicutes*	*Bacillaceae*	0.067	0.097	0.021
